# Neuroligin-2 is ubiquitinated by Nedd4l to control developmental astrocyte morphogenesis

**DOI:** 10.64898/2025.12.15.694023

**Published:** 2025-12-16

**Authors:** Kristina Sakers, Juan J. Ramirez, Nimrod Elazar, Leykashree Nagendren, Erik Soderblom, Cagla Eroglu

**Affiliations:** 1 Department of Cell Biology, Duke University Medical Center, Durham, NC, USA; 2 Howard Hughes Medical Institute, Duke University Medical Center, Durham, NC, USA; 3 Duke Proteomics and Metabolomics Shared Resource and Duke Center for Genomic and Computational Biology, Duke University Medical School, Durham, North Carolina, USA; 4 Department of Neurobiology, Duke University Medical Center, Durham, NC, USA

## Abstract

Astrocytes of the central nervous system have an intricate and highly branched morphology. Proper development of perisynaptic astrocyte processes is necessary for the tripartite synapse development, maturation, and function. However, how astrocyte morphogenesis is controlled, and the specific molecules orchestrating this development are largely unknown. Previously, we identified Neuroligins (NLs) 1–3 as regulators of astrocyte morphogenesis by forming transcellular adhesions with neuronal neurexins. Here, we found that NL2 plays a non-redundant essential role in astrocyte morphogenesis. Through structure and function studies, we identified a WW-binding motif within the NL2 intracellular domain required for astrocyte morphogenesis. Using cell-specific *in vivo* proximity labeling by biotinylation (iBioID), we found that each NL displays distinct protein-protein interactions within astrocytes, and NL2 has cell-type specific interactomes in astrocytes versus neurons. By leveraging this interactome, we conducted *in vitro* and *in vivo* candidate screens and identified WW domain-containing E3 ubiquitin ligase Nedd4l to control astrocyte morphogenesis. Biochemical assays revealed that Nedd4l ubiquitinates and stabilizes NL2, and this ubiquitination step is required for astrocyte morphogenesis. This study shows that Neuroligins have non-overlapping roles in controlling astrocyte growth and uncovers a molecular mechanism of how NL2 mediates astrocyte morphogenesis.

## INTRODUCTION

Protoplasmic astrocytes of the central nervous system (CNS) are easily identified by their unique bush-like morphology, yet the molecular mechanisms that govern how astrocytes develop and their complex structure are largely unknown. Astrocytes tile the brain and extend fine filopodia-like processes into the neuropil(Bushong et al., 2004, 2002), allowing them to play an active role in critical brain functions including synaptogenesis, regulation of synaptic strength, and buffering of neurotransmitters and ions(Farizatto and Baldwin, 2023; Verkhratsky and Nedergaard, 2018). Human astrocytes are disproportionately larger and more complex than rodent astrocytes(Oberheim et al., 2006), suggesting that higher-order species require astrocytes for advanced-level brain function. Consequently, astrocyte dysfunction has been identified across a myriad of neurological diseases(Tian et al., 2010; Rothstein et al., 1995; Preman et al., 2021; Booth et al., 2017; Gutmann et al., 1999). In particular, a recent study found that numerous Alzheimer’s risk genes correlate specifically with astrocyte territory size(Endo et al., 2022). Thus, it is crucial to understand how astrocyte morphogenesis proceeds and which cell biological mechanisms govern this process.

Various signals have emerged as regulators of astrocyte growth, including neuronal-derived secreted factors such as Sonic Hedgehog(Xie et al., 2022) and BDNF(Holt et al., 2019), actin-binding proteins including Ezrin(Endo et al., 2022; Lavialle et al., 2011) and Profilin-1(Molotkov et al., 2013), and numerous cell-adhesion molecules(Baldwin et al., 2023). Amongst the latter, Neuroligins (NLs) play a critical role in the morphogenesis of astrocytes(Ackerman et al., 2021; Stogsdill et al., 2017), as well as oligodendrocyte precursor cells(Li et al., 2024). NLs are trans-synaptic cell-adhesion proteins that bind to Neurexins to promote synaptic formation(Barrow et al., 2009; Chih et al., 2005; Scheiffele et al., 2000; Shipman and Nicoll, 2012), and function(Babaev et al., 2016; Varoqueaux et al., 2006). There are four NL genes expressed in rodents, with NL 1, 2, and 3 being the predominant family members. A limited number of studies have been conducted to investigate how NLs control the outgrowth and complexity of neuronal processes. In rodent neurons, NL1 dose-dependently controls dendritic morphology, wherein NL1 overexpression increases, and NL1 knockdown decreases, dendritic arborization(Schnell et al., 2014, 2012). In contrast, loss of NL3 results in a more complex dendritic arbor(Xu et al., 2019), suggesting non-overlapping roles for NLs in controlling neuronal morphology. Moreover, neuronal NLs were also found to have non-redundant roles in their control of synaptic function(Chanda et al., 2017; Nguyen et al., 2016). In astrocytes, loss of NLs 1–3 each reduces astrocyte territory and neuropil infiltration size(Stogsdill et al., 2017). However, whether NLs play unique roles in astrocyte morphogenesis and what mechanisms control this process is unclear. NLs are implicated in neurodevelopmental diseases including Autism Spectrum Disorder(Jamain et al., 2003; Parente et al., 2017) and thus, it is imperative to understand NL function across all cell types throughout development.

Functional differences across NLs may stem from divergence in amino acid sequence. Rodent NLs 1–3 have similar extracellular domains (ECD, ~75% similarity) and more disparate intracellular domains (ICD, ~50% similarity). Within the ICD, several amino acid motifs have been shown to direct interactions with specific protein partners, ultimately leading to unique functional consequences such as the specification of inhibitory versus excitatory synapses(Poulopoulos et al., 2009; Shipman et al., 2011; Sumita et al., 2007). One study using chimeric NLs demonstrated that NL function is not solely dependent on Neurexin binding to the ECD, but also requires interactions with intracellular proteins in the cytosol to control synapse-specific functions(Nguyen et al., 2016). Further, post-translational modifications on NL ICDs can influence protein-protein interactions and downstream signaling. For example, NL1 tyrosine phosphorylation at Y782 results in preferential binding to the excitatory presynaptic marker PSD-95 over the inhibitory postsynaptic scaffold protein Gephyrin. PSD95 then recruits α-amino-3-hydroxy-5-methyl-4-isoxazolepropionic acid (AMPA) receptors and controls synaptic firing(Giannone et al., 2013; Letellier et al., 2018). Yet, previous studies have been conducted in a neuronal context, and thus, whether astrocytic NLs have distinct protein-protein interactions and how these interactions control astrocyte development is still unknown.

In this study, we show that NLs 1–3 have non-overlapping roles in controlling astrocyte morphological development. We identify that the WW-binding motif in NL2 is necessary for astrocyte morphogenesis. Using an unbiased proximity-labeling approach *in vivo*, we uncover interaction networks for astrocytic NL1, NL2, and NL3. We also performed proximity-labeling for neuronal NL2 to compare cell-type specific differences in NL2 interactome and function. We show that NLs have distinct interactomes representing unique functions within astrocytes and between astrocytes and neurons. These data revealed numerous WW domain-containing proteins that recognize the WW-binding motifs in NLs. Of these, we identified Nedd4l, a HECT-domain E3 ubiquitin ligase, to control NL2 stability via ubiquitination, which is necessary for astrocyte morphogenesis. Moreover, the loss of Nedd4l in astrocytes results in a stunted astrocyte morphology. Importantly, Nedd4l interacts with NL1 and NL2, but not NL3. Together, this study reveals unique interactions of astrocytic NL2, which reveal a novel mechanism of astrocyte morphological development.

## RESULTS

### Astrocytic Neuroligins 1, 2, and 3 have non-redundant roles in morphogenesis.

**Figure 1:**
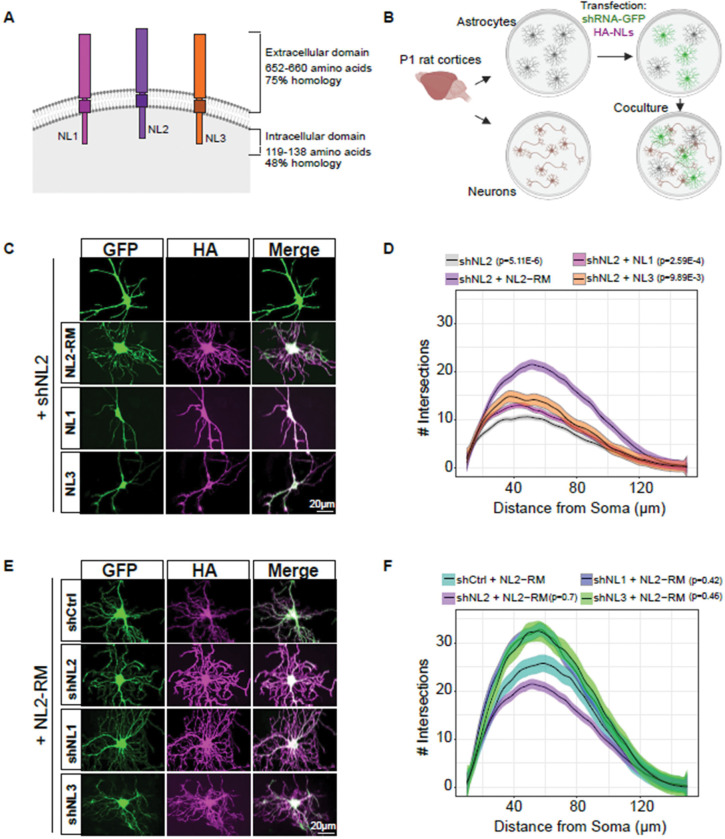
Astrocytic Neuroligins 1, 2, and 3 have non-redundant roles in morphogenesis. A) Cartoon depiction of NLs 1–3. Length and homology refer to mouse sequences. B) Neuron-astrocyte co-culture method to study astrocyte morphogenesis in vitro. P1 rat cortices are used to isolate astrocytes and neurons separately to allow for astrocyte-specific gene manipulation. Astrocytes post-transfection are plated onto neurons for 48h to allow arborization. C) Representative images of astrocytes transfected with shNL2 and either: HA-NL2-RM (Rescue mutant, resistant to shRNA), HA-NL1, or HA-NL3. Scale bars are 20μm. D) Sholl analysis quantification of conditions shown in C. Linear-mixed model followed by ANOVA reveals main effect of condition F(3, 12) = 9.074, p = 0.0021. P-values represent Dunnett’s post-hoc with respect to the shNL2+HA-NL2-RM condition. N = 48–96 cells per condition, across >3 independent culture experiments. Statistical analysis was performed using the biological replicate (independent cultures) N to avoid pseudoreplication artifacts. E) Representative images of astrocytes co-transfected with shRNA against either NL1, NL2, or NL3, and HA-NL2-RM. Scale bars are 20μm. F) Sholl analysis quantification of conditions in E. Linear-mixed model followed by ANOVA reveals trending effect of condition F(3, 11)=3.15, p=0.0686. P-values represent Dunnett’s post-hoc with respect to shCtrl+NL2-RM. N = 53–97 cells per condition, across 3 independent culture experiments. Statistical analysis was performed using the biological replicate (independent cultures) N to avoid pseudoreplication artifacts. Note that the shNL2 + HA-NL2 data is the same across D and F.

NLs 1, 2, and 3 are all expressed throughout development in mouse cortical astrocytes(Boisvert et al., 2018; Clarke et al., 2018; Zhang et al., 2014; Farhy-Tselnicker et al., 2021). Previous work from our lab demonstrated that loss of either NL1, NL2, or NL3 from cortical astrocytes significantly reduces astrocyte complexity. Moreover, the loss of all three NLs simultaneously results in an additive reduction of complexity(Stogsdill et al., 2017), indicating that these proteins play distinct roles during astrocyte development. NLs have similar ECD amino acid sequences yet differ substantially in their intracellular domains (Fig 1A). Considering the differences in amino acid sequence and the combinatorial deficit in astrocyte complexity, we analyzed whether NLs have redundant roles in astrocyte morphogenesis. To address this question, we used an optimized and validated *in vitro* astrocyte-neuron co-culture method to quantify astrocyte branch complexity via Sholl analysis(Stogsdill et al., 2017; Baldwin et al., 2021; Tan et al., 2023) (Fig 1B). Previously, we showed that astrocytes require neuronal contact to develop a highly branched morphology(Baldwin et al., 2021; Stogsdill et al., 2017). In this system, we can manipulate astrocyte gene expression without affecting the neurons. We focused on NL2 because the loss of NL2 affects astrocyte morphogenesis *in vivo* at both early and late developmental time points, suggesting a critical role for astrocytic NL2. We transfected astrocytes with a plasmid expressing an shRNA for *Nlgn2* (shNL2) which has been extensively used and tested for off-target effects(Stogsdill et al., 2017; Chih et al., 2006; Singh et al., 2016). This plasmid also encodes CAG-GFP to visualize transfected cells. Loss of *Nlgn2* in astrocytes causes a substantial reduction in astrocyte morphology when co-cultured with neurons (Fig S1A, B; Fig 1C, D). To provide another line of evidence for the role of Nlgn2 in astrocyte morphogenesis and address potentially confounding results due to shRNA toxicity(Goel and Ploski, 2022), we designed and tested gRNAs targeting rat *Nlgn2* (Fig S1C). Two of three of the gRNAs targeting *Nlgn2* were able to cause a translation-disrupting frameshift mutation, reflected by the loss of GFP expression downstream of the gRNA and PAM sequences (see Methods) (Fig S1D). We expressed these two gRNAs in primary rat astrocytes, along with *Staphylococcus aureus* Cas9 (SaCas9)(Hunker and Zweifel, 2020), under the control of the GfaABC1D promoter(Lee et al., 2008) and found that both gRNAs significantly reduced the morphological complexity in transfected astrocytes cultured with WT neurons, compared to the empty vector control (Fig S1E, F). Thus, these data further support that our Nlgn2 shRNA is specific, and the morphological phenotype cannot be explained by cytotoxicity.

Next we tested whether the stunted morphological phenotype of *Nlgn2* knockdown astrocytes can be rescued by the co-expression of an HA-tagged NL2 that has been modified to be shNL2 resistant (HA-NL2-RM)(Stogsdill et al., 2017) or via the co-expression of HA-tagged NL1 or NL3 with shNL2. Interestingly, HA-tagged NL1 or NL3 did not rescue the astrocyte morphology (Fig 1C, D). In contrast, co-expression of HA-NL2 with either shNL1 or shNL3 curiously resulted in a higher number of maximum intersections in the astrocyte branches compared to a scrambled shRNA control (shCtrl) (Fig 1E, F). Together, these data show that NLs 1, 2, and 3 do not have redundant roles in controlling astrocyte morphogenesis. Moreover, these findings suggest that NL2 is indispensable in astrocyte morphological development.

### The intracellular WW-binding motifs of Neuroligins uniquely regulate astrocyte morphology

**Figure 2:**
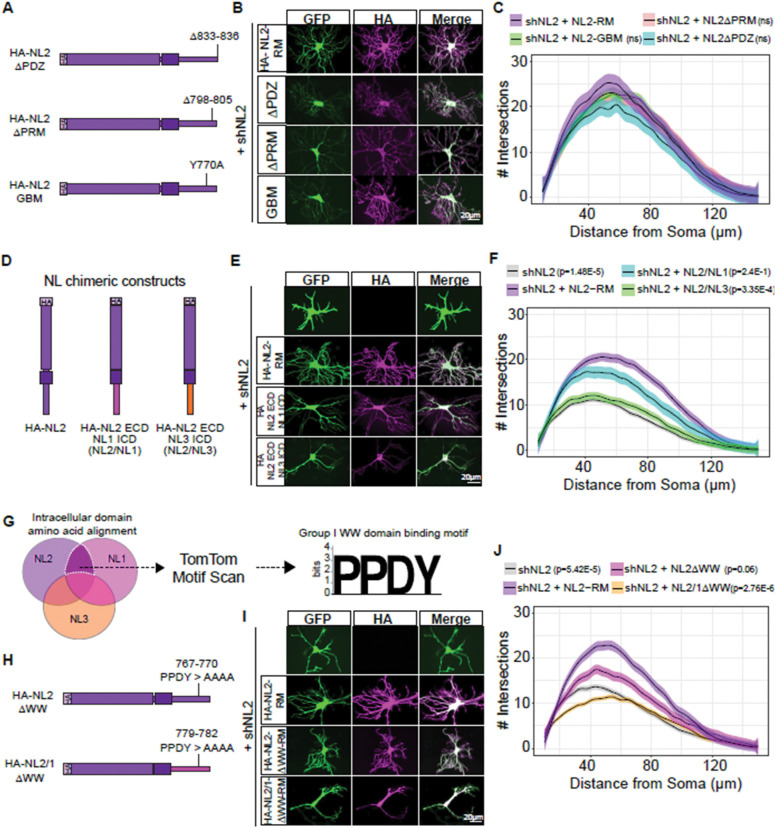
The intracellular WW-binding motifs of Neuroligins uniquely regulate astrocyte morphology. A) Cartoon of NL2 intracellular domain mutants. B) Representative images of astrocytes co-transfected with shNL2 and either HA-NL2-RM (Rescue Mutant, resistant to shRNA) or mutants lacking known motifs. Scale bars are 20μm. C) Sholl analysis quantification of the conditions in D. Linear-mixed model followed by ANOVA reveals no effect of condition F(3, 8)=0.32, p=0.81. NS = not significant in Dunnett’s post-hoc. N=36–46 cells per condition, across 3 independent culture experiments. Statistical analysis was performed using the biological replicate (independent cultures) N to avoid pseudoreplication artifacts. D) Cartoon of NL2-ECD and NL1 or 3 ICD chimeric constructs. E) Representative images of astrocytes transfected with shNL2 and either HA-NL2-RM (Rescue mutant, resistant to shRNA), or chimeric proteins: HA-NL2(ECD)-NL1(ICD), HA-NL2(ECD)-NL3(ICD). Scale bars are 20μm. F) Sholl analysis quantification of conditions in B. Linear-mixed model followed by ANOVA reveals the main effect of condition F(3,12)=8.8, p=0.0023. P-values represent Dunnett’s post-hoc with respect to shNL2+NL2-RM. N=57–97 cells per condition, across 4 independent culture experiments. Statistical analysis was performed using the biological replicate (independent cultures) N to avoid pseudoreplication artifacts. G) Venn diagram depicting the overlap of sequences in NL1, NL2, and NL3 ICDs used for motif scanning by TomTom. We identified the motif PPDY which is common between NL1 and NL2 but absent in NL3. H) Cartoon of NL2ΔWW and NL2/1ΔWW mutant constructs. I) Representative images of astrocytes transfected with shNL2 and HA-NL2-RM-ΔWW or shNL2 and HA-NL2/1-ΔWW. Scale bars are 20μm. J) Sholl analysis quantification of conditions in F. Linear-mixed model ANOVA reveals the main effect of condition F(3, 13)=9.9, p=0.0011. P-values represent Dunnett’s posthoc with respect to shNL2+NL2-RM. N = 71–84 cells per condition, across 4 independent culture experiments. Statistical analysis was performed using the biological replicate (independent cultures) N to avoid pseudoreplication artifacts.

Previous work has revealed unique roles for the NL ICD in controlling synaptic transmission(Nguyen et al., 2016). Thus, we hypothesized that the ICDs of NLs may underlie their distinct roles in controlling astrocyte morphogenesis. NLs contain multiple protein motifs in their ICDs (Fig S2A) that regulate binding to synaptic scaffolding molecules, including Gephyrin and Collybistin(Poulopoulos et al., 2009) which are necessary for inhibitory synapse specification, and PSD-95(Irie et al., 1997), which organizes excitatory postsynaptic sites. We wondered if any of these motifs in the NL2 ICD are necessary for astrocyte morphology. We used mutagenesis to either delete the NL2 PDZ binding domain (NL2 ΔPDZ, deletion of amino acids 833–836), or the NL2 polyproline-rich motif, which activates Collybistin (NL2 ΔPRM, deletion of amino acids 798–805) or mutate NL2 Gephyrin binding residue (NL2 GBM, Y770A) (Fig 2A). We tested whether these mutant constructs could rescue the shNL2 morphological deficits in our co-culture system. These mutants do not alter NL2 localization, as all mutants were trafficked to the cell surface (Fig S2B). Surprisingly, all three mutants were able to rescue the morphological deficits in shNL2 astrocytes (Fig 2B-C), suggesting that potential interactions with Gephyrin, Collybistin, and PSD-95 are dispensable for NL2 control of astrocyte morphology.

To identify the regions of the NL2 ICD that are required for astrocyte morphogenesis, we used previously validated(Nguyen et al., 2016) NL2 chimeric constructs to investigate whether replacing the NL2 ICD with either the NL1 or the NL3 ICD is sufficient to rescue the loss of NL2 in astrocytes (Fig 2D). Using astrocyte-neuron co-cultures, we expressed the NL chimeras in astrocytes in the presence of shNL2 and quantified astrocyte complexity (Fig 2E). While the NL2 ECD/NL1 ICD condition resulted in more complex astrocytes than shNL2 alone, the astrocytes transfected with NL2 ECD/NL3 ICD were identical to shNL2 alone (Fig 2F). This finding suggests that NL1 and NL2 ICD share a common amino acid sequence necessary for controlling astrocyte morphogenesis, which is absent from the NL3 ICD.

To pinpoint the interaction motif in the NL2 ICD that is required for astrocyte morphogenesis, we aligned the intracellular domain amino acid sequences of NL1–3 (Fig 2G, Fig S2A) and isolated motifs that were present in both NL1 and NL2, but absent from NL3. Using TomTom motif scanning(Gupta et al., 2007), we found the sequence PPDY – a Group I WW domain binding motif, known to have the consensus sequence of PPxY(Kasanov et al., 2001; Pirozzi et al., 1997), to fit our criterion. When we mutated the PPDY motif of NL2 ICD to four alanines, this mutant could no longer fully support astrocyte morphogenesis (Fig 2H-J). Similarly, mutation of the PPDY (PPDY > AAAA) in the NL2 ECD/NL1 ICD chimera completely ablated the morphogenesis rescue previously seen for the non-mutated chimera (Fig 2H-J). Together, these data suggest that NL2 controls astrocyte morphogenesis through interactions mediated via the PPDY motif in its ICD.

### *In vivo* BioID reveals unique interactions of astrocytic Neuroligins, including WW domain-containing proteins

**Figure 3:**
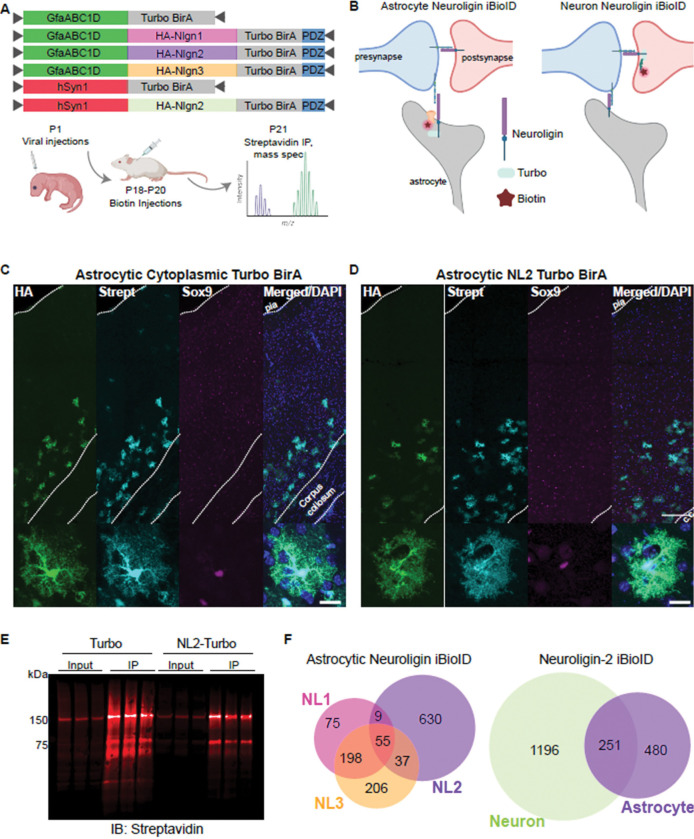
*In vivo* Neuroligin BioID. A) Schematic of AAV constructs and the experimental strategy of in vivo BioID to identify NL interactome *in vivo*. Each biological replicate contained two pooled cortices, 3 biological replicates per condition. B) Cartoon depiction of Neuroligin iBioID constructs in astrocytes or neurons. C-D). Representative cortical images of Astrocytic Turbo BirA (C) or Astrocytic NL2 Turbo BirA (D) injected animals. Stained with Streptavidin-594 (Strept), HA and Sox9 to determine astrocyte specificity. Scale bars: column image = 100μm; inset = 20μm. E) Representative western blot of iBioID immunoprecipitation from astrocytic Turbo vs NL2-Turbo animals demonstrates successful enrichment of biotinylated proteins in immunoprecipitation. Input is 2% of total cortex homogenate. IP is 15% of the eluate sample. Prominent bands are expected at approximately 150 and 75kDa which represent mitochondrial carboxylases(Kirkeby et al., 1993) [blot is overexposed to show this in the input lanes]. Resulting ‘smear’ in IP lanes indicated various-sized proteins that have been biotinylated near the bait protein. F) Venn diagram of protein interactors across astrocytic NL1, NL2, and NL3 and astrocytic versus neuronal NL2.

PPxY motifs are recognized by Group I WW domain-containing proteins(Kato et al., 2004, 2002). WW domains are around 40 amino acids, with two flanking conserved tryptophan (single amino acid code: W) residues(Sudol and Hunter, 2000). In the human proteome, there are over 50 proteins that contain a WW domain, with many proteins containing multiple WW domains. To identify which WW domain-containing protein binds NL1 and NL2, we used *in vivo* BioID (iBioID). This approach(Roux et al., 2012) uses a biotin ligase, Turbo BirA, fused to a bait protein to identify all proteins interacting with the bait, within 10nm(Branon et al., 2018). We made C-terminal fusions of Turbo BirA to NL1, 2, or 3 and virally expressed these constructs under the control of the astrocyte promoter GfaABC1D(Lee et al., 2008) (Fig 3A-D). To investigate cell-type specific differences in NL2 interactome, we also expressed NL2-Turbo BirA under the control of the neuronal promoter hSyn1 (Fig 3A-B). As controls, we expressed soluble TurboID in astrocytes or neurons, which will biotinylate the repertoire of intracellular proteins in each cell-type. We chose to use a cytosol-localized control, as NLs are trafficked throughout many cellular compartments(Kang et al., 2014; Binda et al., 2019; Halff et al., 2019; Xie et al., 2023; Xu et al., 2024), and we aimed to capture all physiologically relevant interactors, not just those at the plasma membrane. We confirmed that the NL2-Turbo BirA constructs were properly localized to the cell membrane in cultured astrocytes (Fig S3A) and they could rescue the morphological deficits of shNL2 astrocytes (Fig S3B, C), thus indicating that NL2 functioned as expected. However, we found that placing the Turbo BirA after the PDZ binding motif in the NL2 C-terminal diminished the biotinylation activity of the enzyme. Thus, we moved the PDZ domain after the TurboID. We could see robust biotinylation after moving the Turbo BirA to just before the PDZ binding motif (Fig S3B) and proceeded with this placement of the TurboID (before the PDZ binding motif) for all NL constructs (Fig 3A).

We injected perinatal mice (P0-P1) intracortically with AAVs expressing the NL-Turbo BirA fusions, or the cell-type-specific soluble BirA control. After three days of biotin injection (P18-P20), we collected P21 mouse cortices and isolated biotinylated proteins by streptavidin immunoprecipitation (Figs 3A, E). We chose P21 as an endpoint because NL2 loss in developing astrocytes results in significantly smaller astrocyte territories with concomitant deficits in synapse formation and function at P21(Stogsdill et al., 2017). We confirmed that our viral vectors were cell-type-specific by staining for the astrocyte-specific marker Sox9 (Fig 3C-D) or the neuronal-specific marker NeuN (Fig S3D), and both induced cell-type-specific biotinylation (visualized by Streptavidin-594 antibody staining) *in vivo*. Mass spectrometry identification and quantification of the biotinylated proteins (Fig S4 A-D, Dataset S1) revealed substantial differences in the putative interactomes of astrocytic NL1–3 (Fig 3F, Dataset S1), when normalized to their respective soluble TurboID controls. Similarly, there were substantial differences between proteins proximal to neuronal versus astrocytic NL2 (Fig 3F, Dataset S1).

**Figure 4:**
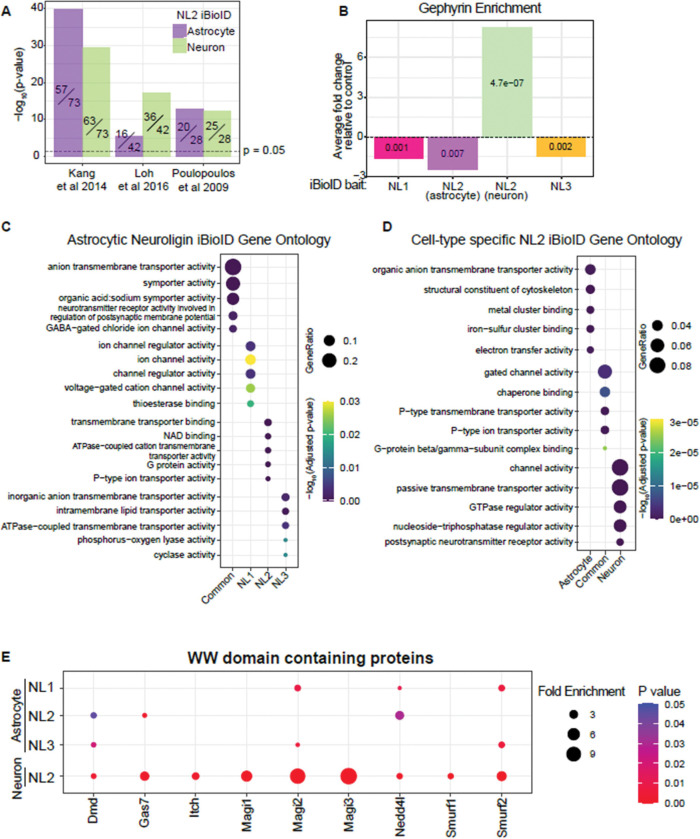
Neuroligin iBioID reveals novel intracellular domain interacting proteins. A) Enrichment of NL2 iBioID datasets with previously published datasets of NL2-interacting proteins. P-values are the result of Fisher’s exact test. The number on the bars indicates the number of proteins from appropriate NL2 iBioID that are found in the published dataset. B) Gephyrin enrichment with each NL iBioID. Enrichment is defined as fold change bait / cytosolic control (Turbo BirA). P values are the results of t-tests within each experiment. C) Top 5 Gene Ontology Biological Processes categories for each NL BioID. Common indicates proteins found in astrocytic NL1, NL2, and NL3 iBioID. The complete gene ontology list can be found in Datset S2. GeneRatio indicates number of gene names (from the input iBioID protein list) in the GO category, out of all gene names in that category. Input for the analysis was proteins from iBioID lists that were at least 1.5 fold enriched at a p-value of <0.05, compared to respective controls. D) Top 5 Gene Ontology Biological Processes categories for each Neuronal or Astrocytic NL2 BioID. Common indicates proteins found in both astrocytic NL2 and neuronal NL2 iBioID. The complete gene ontology list can be found in Dataset S2. GeneRatio indicates number of gene names (from the input iBioID protein list) in the GO category, out of all gene names in that category. Input for the analysis was proteins from iBioID lists that were at least 1.5 fold enriched at a p-value of <0.05, compared to respective controls. E) All identified WW-binding protein interactors in iBioID experiments. Fold change is the enrichment of the interaction with the bait compared to cytosolic Turbo BirA alone, per cell type. P-value represents a two-tailed T-test compared to soluble Turbo BirA, per cell type. Input for the analysis was proteins from iBioID lists that were at least 1.5 fold enriched at a p-value of <0.05, compared to respective controls.

We bioinformatically compared our data to previously published NL2 interactome data (identified via NL2 co-immunoprecipitation in adult mouse whole brain(Kang et al., 2014), *in vitro* extracellular NL2 APEX proximity proteomics(Loh et al., 2016), and a yeast two-hybrid screen of the NL2 ICD(Poulopoulos et al., 2009)) and found a significant overlap with our datasets for each of the previously published data (Fig 4A). We also found noticeable differences between the astrocytic and neuronal interactome of NL2. For example, neuronal NL2 is well characterized to interact with the inhibitory synapse scaffold protein, Gephyrin(Poulopoulos et al., 2009), which we detected to be highly enriched in the neuronal NL2 iBioID interactome (Fig 4B, Dataset S1). However, even though Gepyhrin is present in astrocytes at both the mRNA(Zhang et al., 2014) and protein levels (Dataset S1), we did not detect a significant enrichment of Gepyhrin in the astrocytic NL2 iBioID (Fig 4B, Dataset S1). This result strongly suggests cell-type-specific regulation of protein-protein interactions of NL2 and is consistent with our finding that Gephyrin interaction with NL2 is not necessary for astrocyte morphogenesis (Fig 2A, B).

Gene ontology analysis of the unique proximity interactomes across astrocytic NLs suggests distinct functions of NLs in astrocytes (Fig 4C) and of astrocytic vs neuronal NL2 (Fig 4D). For example, while all astrocytic NLs interact with ion channels and solute transporters, astrocytic NL1 alone interacts with proteins involved in epigenetic modifications, while astrocytic NL2 proximal proteins appear to bind phospholipids. These data support our earlier findings, showing that NLs have non-redundant roles in astrocytes and that these differences likely extend beyond the control of astrocyte morphogenesis. Further, gene ontology analysis of NL2 proximal proteins between neurons and astrocytes reveals functional differences. Interestingly, only astrocytic NL2 interacts with proteins that bind actin filaments (Fig 4D, S4E), which are abundant in perisynaptic astrocyte processes(Soto et al., 2023).

The putative NL2 interactome we identified provided us with the means to determine which nearby proteins could recognize the PPxY motif in NL2 and thus participate in NL2-controlled astrocyte morphogenesis. To do so, we analyzed WW domain-containing proteins across all datasets that were significantly enriched with the bait protein, compared to the cytoplasmic control. Across the 4 iBioID experiments, we found 9 WW domain-containing proteins within NL interactomes (Fig 4E). The WW domain-containing proteins within the NL interactomes varied across NL isoform and cell type. Even though NL1 and NL2 have identical PPxY motifs (PPDY, Fig 2G), there was minimal overlap in WW domain-containing proteins that interacted with both. This finding suggests that the interactions of NLs with WW domain-containing proteins in vivo are not solely determined by the presence of the PPxY motif, and that additional protein-protein interaction motifs in the ICD may confer specificity to these interactions. Furthermore, the utility of including NL3 as a bait in our BioID experiments served as an informative control for the PPxY motif, as NL3 contains the LPxY motif, which is only weakly bound by WW proteins, providing a strong comparative reference for motif-dependent interactions.

### The WW domain-containing E3 ubiquitin ligase Nedd4l is necessary for astrocyte morphogenesis, but not synaptogenesis

The binding of WW domains to WW motifs has various physiological effects in the cell including cellular growth(Lee et al., 2020), endocytosis of membrane proteins(Totland et al., 2017), and transcription(Sudol et al., 2001). Importantly, WW domain-containing proteins and their interactions with WW motifs have been implicated in various diseases, including the neurodevelopmental disorder Rett Syndrome(Rodrigues et al., 2020), Muscular Dystrophy(Ilsley et al., 2002), and hypertension(Minegishi et al., 2016). We observed three significantly enriched WW domain-containing protein interactors of astrocytic NL2, Dystrophin (*Dmd*), growth arrest-specific protein 7 (*Gas7*), and neural precursor cell expressed developmentally downregulated 4-like protein (*Nedd4l*, also called Nedd4–2) (Fig 4E). We chose to focus on Nedd4l from these results for two reasons. First, both NL1 and NL2 PPDY motifs are necessary for astrocyte morphogenesis (Fig 2I-J), and Nedd4l is enriched in astrocytic NL1 and NL2, but not NL3 (Fig 4E) proximity proteomes. Second, Nedd4l has a known role in regulating astrocyte membrane proteins. For example, Nedd4l controls the abundance of the potassium channel Kir4.1, the gap junction protein, Connexin-43(Altas et al., 2024; Liu et al., 2021) as well as the abundance and surface localization of the glutamate transporter Glt-1(Zhang et al., 2017), via ubiquitination.

**Figure 5:**
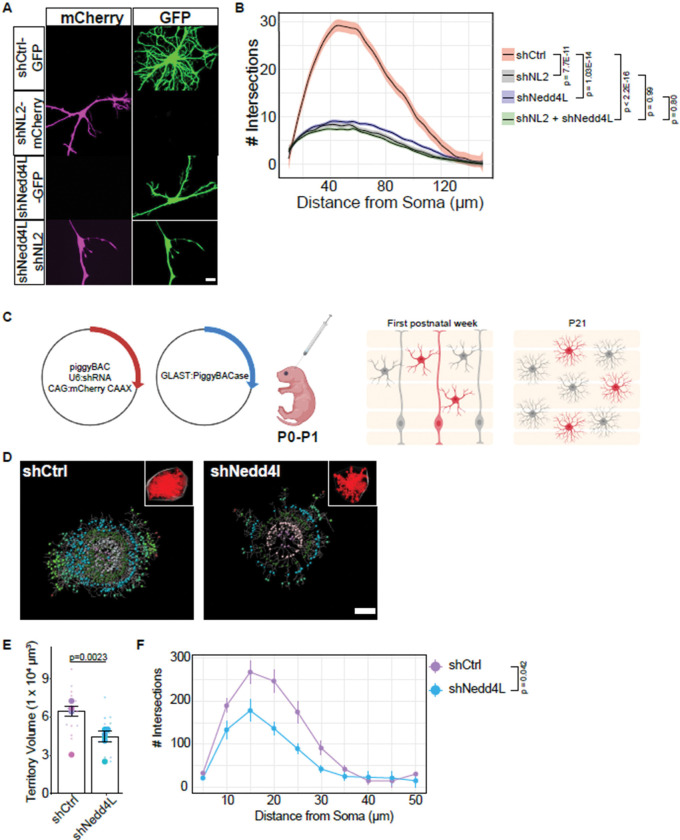
WW domain-containing proteins are necessary for astrocyte morphogenesis in vitro. A) Representative images of astrocytes transfected with either a control shRNA (scrambled NL2 shRNA), an shRNA targeting Nedd4l (also expressing GFP) or shNL2 (also expressing mCherry), or in combination. Astrocytes are cultured on top of neurons (not labeled). Scale bar is 20μm. B) Sholl analysis of the conditions in A. Linear-mixed model ANOVA reveals the main effect of condition F(3, 13)=38.104, p=1.05E-6. P-values on the graph represent Tukey’s posthoc comparison to shCtrl. N = 60–100 cells per condition, across 4 independent culture experiments. Statistical analysis was performed using the biological replicate (independent cultures) N to avoid pseudoreplication artifacts. C) Cartoon of postnatal astrocyte labeling by electroporation (PALE). Two plasmids (U6:shRNA and CAG:mCherry CAAX flanked by piggyBAC transposons, and GLAST: piggyBACase transposase) are electroporated into late P0 or early P1 mouse lateral ventricles. Brains are collected at P21 and stained for mCherry to visualize astrocyte territories. D) Imaris reconstructions of *in vivo* astrocyte territory volumes (inset) and 3D Sholl analysis of mouse cortical astrocytes. ‘Filament’ cytoskeletal structure is shown with grey lines, while spheres indicate 3D Sholl concentric circles [different colors indicate different distances from the nucleus]. Scale bars = 10μm. E) Territory volume super plot quantification of the conditions in C. Large dots on graph = average per animal. Smaller dots = individual cells. Data were analyzed using a nested ANOVA; main effect of condition, F(1, 8) = 11.95, p=0.0023. The same cells were measured for territory volume and Sholl analysis. N=14–18 cells across 4–7 animals (biological replicates) per condition. Statistical analysis was performed on the biological replicate number and not the number of cells to avoid pseudoreplication artifacts. F) 3D Sholl analysis. Linear mixed model ANOVA F(1,9)=5.59, p=0.0424. The same cells were measured for territory volume and Sholl analysis. N=14–18 cells across 4–7 animals (biological replicates) per condition. Statistical analysis was performed on the biological replicate number and not the number of cells to avoid pseudoreplication artifacts.

To investigate if Nedd4l is necessary for astrocyte morphogenesis, we designed and validated an shRNA (Figs 5A, S5A-B) targeting *Nedd4l*. We expressed this shRNA in astrocytes to determine how the loss of Nedd4l impacts the morphological complexity of astrocytes cultured with wild-type neurons. Strikingly, we observed a severe reduction in astrocyte morphological complexity in shNedd4l-transfected astrocytes, compared to control (Fig 5A-B). Co-knockdown of *Nedd4l* and *Nlgn2* (using a plasmid that also encodes mCherry) did not further reduce astrocyte complexity. These findings suggest that Nedd4l and NL2 might work in the same pathway (Fig 5B).

Next, we tested whether Nedd4l is necessary for astrocyte morphogenesis *in vivo*. To achieve genetically targeted and sparsely labeled astrocytes in the mouse cortex, we used postnatal astrocyte labeling by electroporation (PALE)(Baldwin et al., 2021; Stogsdill et al., 2017). In this method, plasmid DNA expressing a shRNA with CAG:mCherry-CAAX and a GLAST promoter-driven piggyBac enzyme are injected into P0-P1 mouse lateral ventricles and then electroporated into the cortex. This technique results in the uptake of the plasmid by radial glia and subsequent astrocyte labeling after differentiation (Fig 5C). Using this technique, we previously found that loss of astrocytic NL2, using both shRNA targeting *Nlgn2* or a floxed allele with astrocytic Cre expression, results in a significantly stunted astrocyte territory volume and branch complexity(Stogsdill et al., 2017). We used confocal imaging of the entire astrocyte in 100μm tissue sections and reconstructed the astrocyte volume using Imaris software. Quantification of the astrocyte territory volume revealed a significant reduction in shNedd4l astrocytes, compared to a scrambled shRNA control (shCtrl) (Fig 5D-E). Additionally, quantification of the astrocyte complexity using *in vivo* Sholl analysis revealed a significant decrease in complexity of Nedd4l knockdown astrocytes (Fig 5F). These data suggest that astrocyte morphogenesis is controlled by Nedd4l at both the level of territory size and branch complexity.

We previously showed that loss of *Nlgn2* in sparsely labeled astrocytes in the mouse V1 cortex results in reduced excitatory synaptogenesis in the surrounding neuropil. Thus, next, we tested if Nedd4l loss also affects synapse density by using PALE to knock down *Nedd4l* in sparse astrocytes in the mouse V1 cortex. At P21, we stained cortical sections for presynaptic Bassoon, and postsynaptic PSD95 and Gephyrin. Using a combination of these antibodies, we quantified both excitatory (closely apposed Bassoon and PSD95) and inhibitory (Bassoon and Gephyrin) synapses in the same sections (Fig S6B). We used the mCherry signal from the piggyBAC vector (Fig 5C) to define perturbed astrocyte territories. Using open-source SynBot software(Savage et al., 2024), we quantified the synaptic density within labeled astrocytes and outside of labeled astrocytes (Fig S6A-B). These data revealed no significant differences in synapse density in *Nedd4l*-knockdown astrocytes, *in vivo* (Fig S6C-D). This data suggests Nedd4l and its interaction with NL2 controls astrocyte morphological development independently from the role of astrocytic NL2 in synaptogenesis. Thus, the WW domain-containing protein Nedd4l is necessary for astrocyte morphogenesis, but not for astrocyte-dependent synaptogenesis.

### The PPDY motif in Neuroligin-2 is sufficient for Nedd4l interaction

**Figure 6:**
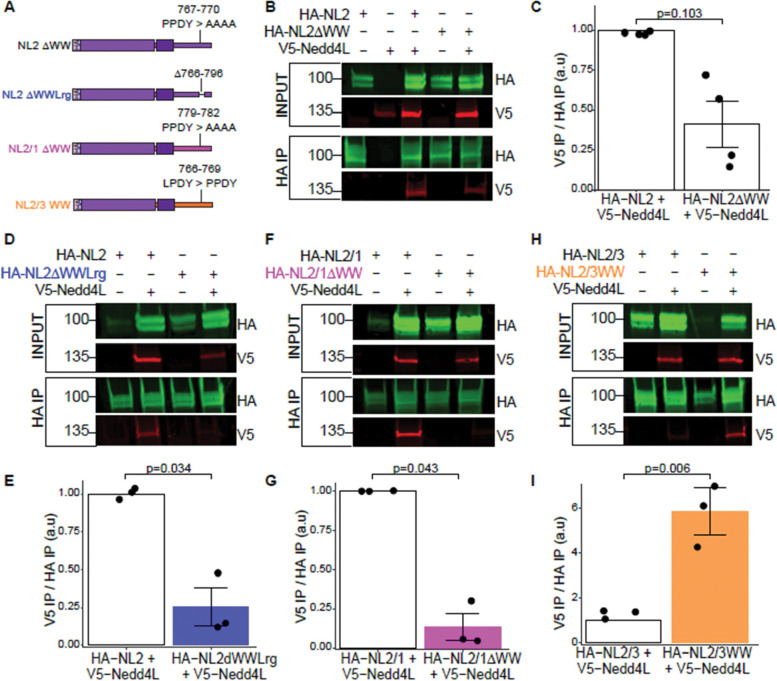
Nedd4l binding to Neuroligins is modulated by intracellular PPxY motifs. A) Cartoon of NL2 and NL2-ICD chimeric constructs. Numbers indicate amino acid positions in mouse sequence. B) Western blots from HEK cell lysates transfected with HA-NL2 or HA-NL2ΔWW with or without V5-Nedd4l constructs. Co-immunoprecipitation (IP) of V5-Nedd4l was detected with NL2-HA constructs using Li-Cor fluorescent imaging. C) Densitometry quantification of IP signal from conditions in B. N=4 biological replicates. P-values represent results of paired, Student’s t-test. D) Western blots from HEK cell lysates transfected with HA-NL2 or HA-NL2ΔWWLrg (Deletion of amino acids 766–796) with or without V5-Nedd4l constructs. Co-immunoprecipitation (IP) of V5-Nedd4l was detected with NL2-HA constructs using Li-Cor fluorescent imaging. E) Densitometry quantification of IP signal from conditions in D. N=3 biological replicates (independent culture experiments). P-values represent results of paired, Student’s t-test. F) Western blots from HEK cell lysates transfected with HA-NL2/1 or HA-NL2/1ΔWW with or without V5-Nedd4l constructs. G) Densitometry quantification of IP signal from conditions in F. N=3 biological replicates. P-values represent results of paired, Student’s t-test. H) Western blots from HEK cell lysates transfected with HA-NL2/3 or HA-NL2/3-WW with or without V5-Nedd4l constructs. I) Densitometry quantification of IP signal from conditions in H. N=3 biological replicates. P-values represent results of paired, Student’s t-test. (B, D, F, H) All western blots were run at least 3 times with independent samples. Note that Nedd4l (V5) blots were run separately (eluate divided evenly) to avoid bleed-through from the strong HA signal.

The Nedd4 family is part of Group I WW binding proteins, which exhibit preferential binding to PPxY motifs and only weakly bind to LPxY(Hatstat et al., 2021). In the absence of a PPxY containing substrate, Nedd4l is known to bind its own LPxY motif, autoubiquitinate and degrade(Bruce et al., 2008). Because NL1 and NL2 have a PPxY motif, and NL3 has an LPxY motif (Fig 2G; Fig S2B), we wondered if Nedd4l preferentially and directly interacted with NL1 and NL2 ICDs but not the NL3 ICD. To test this, we used either the wildtype NL2 or NL2 ECD/NL1 ICD chimeras (Fig 2D) and mutated the NL2 ICD and NL1 ICD PPDY motifs to alanines to abolish the WW domain-containing motif, as described earlier (Fig 2H-J; Fig 6A). Conversely, we also mutated the NL3 ICD LPDY motif to PPDY to mimic NL1 and NL2 ICDs. All chimeric constructs contained the NL2 ECD to rule out the effects of sequence divergence in NL ECDs.

Next, we co-expressed HA-tagged NL2 and NL2 chimeras with V5-tagged Nedd4l in HEK 293T cells and co-immunoprecipitated NLs using anti-HA tag magnetic beads. We found that Nedd4l co-immunoprecipitated with wildtype NL2 (Fig 6B); however, ablation of the PPDY motif in NL2 ICD (NL2ΔWW, Fig 6A) resulted in a substantial, yet variable, reduction of Nedd4l co-immunoprecipitation (Fig 6B, C). A previous study investigating the interaction of NL2 and the WW domain-containing protein S-SCAM (*Magi2*) found that a longer stretch of amino acids 766–796, which contains the PPDY motif, in the NL2 ICD is necessary for direct binding of S-SCAM to NL2(Sumita et al., 2007). Thus, we next deleted these amino acids from the NL2 ICD (NL2ΔWWLrg, Fig 6A) and performed co-immunoprecipitation with Nedd4l. Loss of this sequence in NL2 ICD completely abolished the interaction with Nedd4l (Fig 6D-E), demonstrating that this is a critical region in NL2 ICD containing the PPDY motif that mediates Nedd4l binding.

Ablation of the PPDY motif in NL1 ICD (NL2/1ΔWW, Fig 6A), however, abolished the interaction with Nedd4l entirely, further showing the importance of the PPDY motif in NL-Nedd4l interactions (Fig 6F-G). As expected, we found that Nedd4l does not interact with wild-type NL3 ICD (Fig 6H). However, strikingly, mutating the LPDY motif of NL3 ICD to PPDY (NL2/3WW, Fig 6A, H-I) was sufficient to induce an interaction between Nedd4l and NL3 ICD. These results reveal that the PPDY is sufficient for Nedd4l interactions with NL ICDs. Additional sequences in the NL2 ICD between amino acids 766–796 flanking the PPDY motif are also involved in NL2/Nedd4l binding.

### Nedd4l ubiquitinates Neuroligin-2 at K749, resulting in increased stability of Neuroligin-2

**Figure 7:**
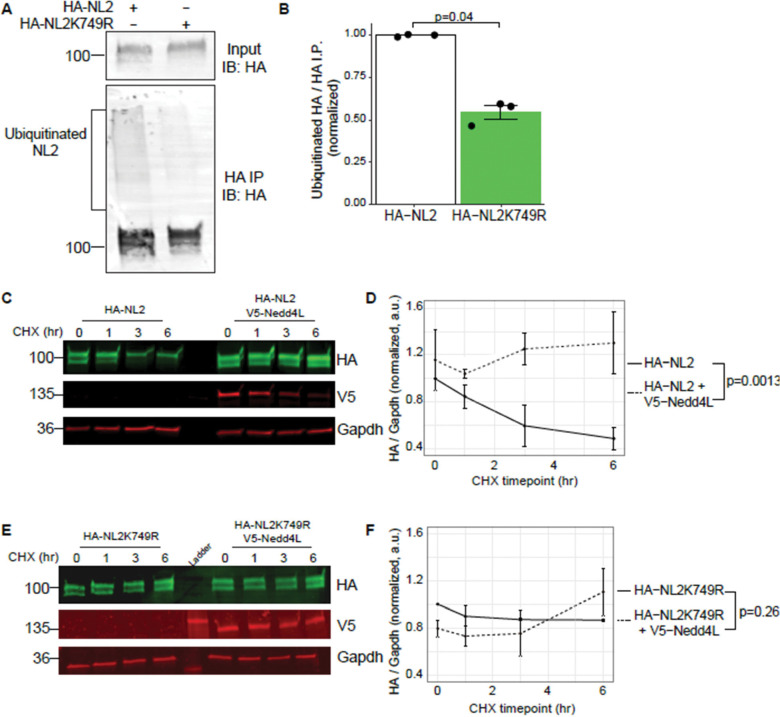
Nedd4l stabilizes Neuroligin-2 via ubiquitination at K749R. A) Western blot from rat primary astrocyte cell lysates and HA-NL2 IPs shows NL2 is ubiquitinated in astrocytes, which is lost when NL2 K749 is mutated to arginine (K749R). Input is 2% of total lysate. B) Quantification of HA ubiquitination signal from conditions in A. The ubiquitinated HA bands >100kDa were quantified via densitometry and normalized to the HA IP band (~100kDa) per biological replicate. N=3 biological replicates (independent culture experiments). P-values represent results of paired, Student’s t-test. C) Western blots from HEK cell lysates transfected with HA-NL2 or HA-NL2 + V5-Nedd4l and treated with CHX. Numbers above the blot indicate hours after CHX treatment. D) Quantification of the conditions in B. Samples were collected on N=3 independent samples. Two-way ANOVA reveals main effect of condition F(1, 16)=15.018, p=0.00134, with no significant effect of time-point F(3, 16)=0.41, p=0.74827 or interaction F(3, 16)=2.242, p=0.123. E) Western blots from HEK cell lysates transfected with HA-NL2K749R or HA-NL2K749R + V5-Nedd4l and treated with CHX. Numbers above the blot indicate hours after CHX treatment. F) Quantification of the conditions in D. Samples were collected on N=3 independent samples. Two-way ANOVA reveals no significant effect of condition F(1, 16)=1.392, p=0.255, time-point F(3, 16)=1.681, p=0.211 or interaction F(3, 16)=2.328, p=0.113.

Nedd4l is an E3 ubiquitin ligase of the HECT domain family which has previously been shown to polyubiquitinate the astrocyte membrane proteins Kir4.1, Connexin-43, and Glt-1, resulting in their degradation(Altas et al., 2024; Liu et al., 2021; Zhang et al., 2017). Previous proteomic studies have identified that the intracellular domain of NL2 is ubiquitinated at lysine 749 (K749)(Na et al., 2012; Wagner et al., 2012) in both mouse and rat brain tissues. However, the function of this posttranslational modification in NL2 is unknown. Thus, next, we investigated whether NL2 is ubiquitinated at K749. To do so, we first blocked proteasomal degradation using the proteasomal inhibitor MG-132. We next heat-denatured lysates to disrupt protein-protein interactions and subsequently immunoprecipitated HA-NL2 from primary rat astrocytes. After running the immunoprecipitated eluates on a western blot, we found that HA-NL2 showed a smear of high molecular weight HA-immunoreactive protein, indicative of polyubiquitinated protein (Fig 7A). We found that we could significantly decrease this polyubiquitination by mutating K749 to R (Arginine), as immunoprecipitation of HA-NL2K749R primarily displayed only one band at the expected molecular weight for NL2 (Fig 7A-B). These data show that NL2 is ubiquitinated in primary astrocytes, which can be blocked by mutating K749.

We next aimed to test if Nedd4l ubiquitinates NL2. Interestingly, while our *Nedd4l* expression construct works well in HEK 293T cells, we were unable to overexpress Nedd4l in primary astrocytes. This may be due to global proteasome disruptions and cytotoxicity due to Nedd4l’s role in astrocyte protein turnover(Altas et al., 2024). To circumvent this, we turned to HEK 293T cells and expressed HA-NL2 with or without V5-Nedd4l co-expression and then probed for GFP-tagged ubiquitination via Western blot with a GFP antibody. We found that, indeed, compared to NL2 alone, co-expression with Nedd4l resulted in a high molecular weight smear above the expected size of NL2 indicating that Nedd4l is sufficient to enhance polyubiquitination of NL2, when compared to NL2 alone (Fig S7A-B). Further, we could reduce this ubiquitination by mutating K749 of NL2 to arginine (K749R). Co-expression of Nedd4l and NL2K749R also did not result in enhanced polyubiquitination of NL2, indicating that K749 is the only ubiquitinated residue in NL2 (Fig S7A-B). Together, these data reveal that Nedd4l is sufficient to ubiquitinate NL2 at K749.

While ubiquitination is typically associated with protein degradation, it can also serve as a posttranslational signal for various cellular processes(Mukhopadhyay and Riezman, 2007). We first tested whether Nedd4l-mediated ubiquitination of NL2 results in the degradation of NL2. We expressed NL2 with or without Nedd4l in HEK cells and collected cell lysates at different time points after cycloheximide (CHX) treatment, which blocks nascent translation. Western blotting of these samples revealed a decrease in HA-NL2 abundance over time, indicating NL2 is degraded (Fig 7C-D). Strikingly, co-expression of Nedd4l with NL2 completely blocked this degradation and stabilized NL2 protein over time (Fig 7C-D). Interestingly, mutation of NL2 lysine 749 to arginine (K749R) was sufficient to block NL2 degradation, with no further effect by the addition of Nedd4l (Fig 7E-F). Taken together, these data reveal that ubiquitination at K749 controls NL2 degradation; however, ubiquitination at K749 by Nedd4l stabilizes NL2 protein and prevents its degradation.

### Ubiquitination of Neuroligin-2 at Lysine 749 is required for astrocyte morphogenesis

**Figure 8:**
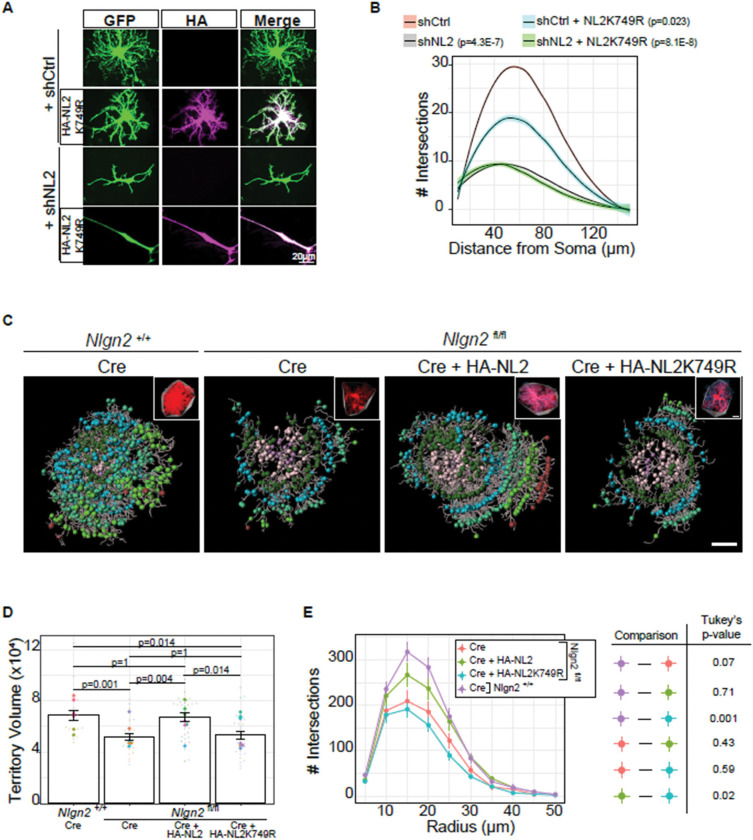
Neuroligin-2 ubiquitination at K749 is critical for astrocyte morphogenesis. A) Representative images of astrocytes transfected with either shCtrl or shNL2 with or without HA-NL2K749R expression. Scale bars are 20μm. B) Sholl analysis quantification of images in J. Linear-mixed model ANOVA reveals the main effect of condition F(3, 8)=13.49, p=0.0017. P-values represent Dunnett’s posthoc with respect to shCtrl. N = 62–80 cells per condition, across 3 independent culture experiments. C) Representative images of reconstructed astrocytes. ‘Filament’ cytoskeletal structure is shown with grey lines, while spheres indicate 3D Sholl concentric circles [different colors indicate different distances from the nucleus]. Insets show the astrocyte signal [tdTomato signal in red; HA signal in cyan] with the convex hull [grey surface lines]. Scale bar = 10μm. D) Territory volume super plot quantification of the conditions in C. Large dots on graph = average per animal. Smaller dots = individual cells. Data were analyzed using Kruskal-Wallis rank sum test: χ^2^ = 19.5, df=3, p=0.00013. P-values on the graph are results of the Wilcoxon rank sum post hoc tests with Holm-Šídák multiple comparison correction. The same cells were measured for territory volume and Sholl analysis. N=21–33 cells across 6–7 animals (biological replicates) per condition. E) 3D Sholl analysis. Linear mixed model ANOVA F(3, 105)=5.46, p=0.0016. P-values in the table represent Tukey’s post hoc tests. The same cells were measured for territory volume and Sholl analysis. N=21–33 cells across 6–7 animals (biological replicates) per condition.

NL2 is ubiquitinated, *in vivo*(Na et al., 2012; Wagner et al., 2012), yet whether ubiquitination at this residue has a functional role is unexplored. Therefore, we next tested if ubiquitination at K749 is necessary for NL2’s control of astrocyte morphogenesis. To do so, we expressed NL2K749R (also mutated to be resistant to shNL2) in primary astrocytes and subsequently co-cultured the astrocytes on top of neurons to quantify astrocyte complexity. Surprisingly, we found that NL2K749R expression significantly reduced astrocyte morphology in a dominant-negative fashion compared to the scrambled shRNA control (Fig 8A-B). NL2K749R was also incapable of any morphological rescue of shNL2 transfected astrocytes (Fig 8A-B).

Finally, we investigated whether NL2K749R is crucial for astrocyte morphogenesis in the developing mouse cortex. In *Nlgn2* homozygous flox animals (*Nlgn2* ^fl/fl^), also carrying a Rosa26:lox-STOP-lox tdTomato allele, we used PALE (Fig 5C), to express Cre to ablate *Nlgn2* in astrocytes, while simultaneously expressing HA-NL2 (WT) or HA-NL2K749R (Fig 8C). At P21, we harvested the brains to analyze astrocyte morphological development via Imaris reconstruction and 3D Sholl analysis. As previously shown(Stogsdill et al., 2017), the knock-out of both copies of *Nlgn2* via Cre expression in astrocytes results in a significantly diminished territory volume, compared to *Nlgn2* ^+/+^ animals (also carrying the Rosa26:lox-STOP-lox tdTomato allele) (Fig 8D). This morphological deficit was rescued by co-expressing an HA-tagged NL2 plasmid in the astrocytes (Fig 8D). Supporting our *in vitro* findings (Fig 8B), co-expression of HA-NL2K749R with Cre in *Nlgn2* ^fl/fl^ astrocytes was not able to rescue the reduction in territory volume, and showed no significant difference between *Nlgn2* ^fl/fl^ astrocytes that received Cre alone (Fig 8D). We did observe a bimodal distribution in the territory volumes of *Nlgn2* ^fl/fl^ astrocytes that received Cre + HA-NL2K749R that was not related to sex or astrocyte layer identity, suggesting an unknown variable influences the functional importance of NL2 ubiquitination status. Further, we found that astrocyte complexity, measured by 3D Sholl analysis (Fig 8E), largely mirrored the territory volume findings in Fig 8D. Altogether, these findings show that ubiquitination of NL2 at K749 is necessary for its role in astrocyte morphogenesis at both the territory volume and branch complexity levels.

## DISCUSSION

Astrocytes play crucial roles in nervous system development and function. Proper astrocyte function relies on their highly branched astrocyte morphology, which is largely controlled by cell-adhesion molecules(Ackerman et al., 2021; Baldwin et al., 2021; Stogsdill et al., 2017; Tan et al., 2023). However, the mechanisms downstream of these cell adhesion molecules are not yet fully understood. Here, we show that NLs are non-redundant in controlling astrocyte morphology due to their unique intracellular protein interactomes. We found a critical motif that regulates Nedd4l interaction and ubiquitination of NL2, which is necessary for NL2-mediated control of astrocyte morphogenesis. Further, we provide *in vivo* astrocytic NL1–3 and neuronal NL2 proximity proteomes, which provide insights into the differences in astrocytic NL function and cell-type-specific functions of NL2 between astrocytes and neurons.

How do NLs uniquely contribute to astrocyte morphogenesis? We found that overexpression of NLs could not compensate for the loss of one another *in vitro*, which may be explained by various reasons. First, NL mRNA expression patterns differ in the developing cortex(Clarke et al., 2018), suggesting that temporal dynamics of NLs may differentially control astrocyte cytoarchitecture. Second, NLs form both homo- and heterodimers, and dimerization is necessary for both localization and function(Poulopoulos et al., 2012; Shipman and Nicoll, 2012). In neurons, only NL1/NL3 and NL1/NL2 heterodimers have been detected(Poulopoulos et al., 2012); however, our results indicate that both NL1/NL2 and NL2/NL3 dimers exist in both astrocytes and neurons, suggesting that cell-type-specific heterodimers may impact NL function during brain development. Lastly, our interactome data reveals disparate protein interactions with NLs which likely contribute to unique astrocytic NL function. Interestingly, gene ontology analysis revealed a significant interaction with actin binding proteins and cytoskeletal related proteins specifically for astrocyte NL2, and minimally for astrocyte NL1 or NL3 or neuronal NL2, suggesting astrocytic NL2 plays a unique role in controlling astrocyte morphology (Dataset S2, Fig S4 E-F). However, it is important to note a few limitations of our iBioID data: first, because the TurboID promiscuously biotinylates proteins within 10nm, interactions with a bait protein (here, NLs) may or may not be due to direct binding and should be validated orthogonally, as we showed with Nedd4l (Fig 6). Second, interactions with NLs may be transient and/or developmentally regulated, and thus, our 3-day biotinylation treatment window may capture both static and transient interactions. Therefore, these data should not be interpreted as a compendium of NL-interacting proteins. Lastly, GFAP promoter activity is highest in the lower layers of the cortex (Fig 3C-D), thus our astrocyte-specific proteomic experiments are largely influenced by lower-layer astrocytes. Nevertheless, the iBioID interactomes generated in this study will be beneficial in elucidating the mechanisms through which NLs control astrocyte structure and function.

In this work, we show that the WW domain-containing protein Nedd4l is critical for astrocyte morphogenesis, and this protein has distinct specificity for each NL, which we validated by direct protein-protein interaction. How do WW domain proteins specify interactions with NLs? Nedd4l preferentially binds PPxY motifs, but can also weakly bind LPxY(Bruce et al., 2008; Hatstat et al., 2021). We identified that NL1 and NL2 contain a PPxY motif in their ICDs, whereas NL3 contains an LPxY motif. This single amino acid change ultimately impacts the binding of Nedd4l to NLs but does not explain the interaction specificity entirely. Only after deleting amino acids 766–796 in NL2, ICD Nedd4l binding is blocked, suggesting a larger surface is critical for the interaction between NL2 and Nedd4l. This may explain why mutation of PPDY to AAAA in the NL2 ICD is still able to restore some of the astrocyte complexity (Fig 2J) and suggests that the WW binding motif in NL2 is not the sole determinant of NL2 function in astrocyte morphogenesis. Moreover, in our iBioID results, Nedd4l is not enriched in NL1 proximal proteome as strongly as NL2 in astrocytes, which may indicate a weaker physiological interaction. Tyrosine phosphorylation in WW motifs is known to occlude binding with WW domains(Chen et al., 1997). The conserved Tyrosine (Y) in the PPxY motif is the same residue that modulates Gephyrin binding (Y770)(Poulopoulos et al., 2009) and is phosphorylated in neuronal NL1 but not neuronal NL2(Letellier et al., 2018), therefore, phosphorylation and/or Gephyrin binding might modulate Nedd4l binding to NLs. Nedd4l interactions with its known targets are controlled through adapter proteins, namely the 14–3-3 family(Lee et al., 2018; Nagaki et al., 2006). Our data reveals that astrocytic NL2 interacts with several of the 14–3-3 family proteins, pointing toward a role of 14–3-3 proteins mediating the Nedd4l-NL2 interaction in astrocytes. Moreover, several WW domain-containing proteins interact with NLs in a cell-type-specific manner (Fig. 4E). Future studies will reveal the cell-type-specific effects of WW domain-containing protein interactions with NLs.

Until this study, the functional consequence of NL2 ubiquitination had not been demonstrated in either astrocytes or neurons. We found that Nedd4l ubiquitinates NL2 at K749. Numerous reports indicate that Nedd4l ubiquitinates its targets, including the astrocytic targets Kir4.1 and Connexin-43(Altas et al., 2024), which results in proteasome-mediated degradation(Liu et al., 2021; Zhang et al., 2017; Zhou et al., 2007). However, here we found that Nedd4l ubiquitination of NL2 results in increased stability of NL2. Interestingly, ubiquitination-mediated protein stabilization has been reported previously for other HECT E3 ubiquitin ligases. Nedd4, a close family member of Nedd4l, has been demonstrated to ubiquitinate another E3 ubiquitin ligase, Mdm2, which results in increased stability of the protein(Xu et al., 2015). We speculate that Nedd4l-mediated stability of NL2 may maintain the surface pool of NL2 and/or overall NL2 abundance, to maintain synaptic contacts with Neurexins. While Nedd4l ubiquitination of NL2 does not lead to protein degradation, our data reveal that NL2K749 ubiquitination can be a degradative signal. In heterologous cells, NL2 protein abundance significantly decreases over time, and blocking ubiquitination at K749 prevents this degradation (Fig 7C-F). A limitation of this experiment is that it was performed in heterologous cells, due to the low expression, possibly resulting from rapid degradation, of astrocytic NL2 in the absence of neurons. Future work using cell-type-specific knock-in or conditional degradation models will be required to test Nedd4l’s role in regulating NL2 stability in astrocytes, *in vivo*. Finally, ubiquitination of NL2 was identified in both mouse and rat brains at lysine K749 via ubiquitin proteomics, but NL1 and NL3 were not(Na et al., 2012; Wagner et al., 2012). We found that blocking ubiquitination at K749 results in a stunted morphological phenotype for NL2-mediated astrocyte morphogenesis, both *in vitro* and *in vivo* (Fig 8A-E). This phenotype may be due to an excess of NL2 in the astrocyte surface, thus leading to improper targeting of perisynaptic astrocyte processes in the neuropil and preventing precise interactions with neuronal neurexins. To this end, understanding how trans-synaptic neurexin binding to NL2 impacts Nedd4l interaction with NL2 and/or ubiquitination of NL2 will be an important future direction. Altogether, this finding underscores the importance of NL2 turnover in the development of astrocyte morphology.

Emerging evidence emphasizes the differential roles of astrocytic versus neuronal NLs. Numerous laboratories have identified a unique physiological role of NLs in various glia including astrocytes(Stogsdill et al., 2017; Ackerman et al., 2021), oligodendrocyte precursor cells(Li et al., 2024), as well as glioma(Venkatesh et al., 2015, 2017). Recently, an article claimed that astrocytic NLs do not control astrocyte morphogenesis or synapse formation(Golf et al., 2025). This study used an astrocyte Cre line and floxed NL alleles to knock out *Nlgns1–3* in the mouse brain, yet analyzed effects at different timepoints than our study. First, the authors demonstrate a decrease in astrocyte-specific ribosome-bound mRNA only at 8 weeks postnatally, but show no difference in mouse cortical astrocyte morphology at P35. We suspect that their incongruous findings are due to incomplete knock-down of NLs early in development, which we have previously shown to be particularly difficult to knock out *in vivo* using the traditional Cre-Lox system(Stogsdill et al., 2017). In this work, to circumvent such issues, we have used carefully validated shRNAs(Chih et al., 2006) *in vitro* and *in vivo*, which have no compounding effect in NL2 cKO astrocytes, indicating no off-target effects(Stogsdill et al., 2017), and we have validated these findings using the CRISPR-Cas9 system (Fig S1C-F) and PALE-mediated rescue of NL2-dependent astrocyte morphogenesis (Fig 8C-E). Further, our study lends insight into how slight perturbations of NL2 abundance, in either direction, derail astrocyte morphology. We find that overexpression of NL2 does not completely rescue the morphological phenotype of shNL2 expressing astrocytes (Fig S1B), suggesting that too much astrocytic NL2 results in an overly adhesive phenotype. This aligns with the dominant negative morphological phenotype of astrocytes expressing NL2 with the K749R mutation (Fig 8A-B), in which we expect too much NL2 protein in the astrocytes due to the lack of proteasome-mediated degradation of NL2 (Figure 7C-D).

In summary, we provide new insight into NL-mediated control of astrocyte morphogenesis and reveal that the WW domain-containing protein, Nedd4l, is required for astrocyte morphogenesis via ubiquitination and stabilization of NL2. Lastly, we show that NL2 ubiquitination is critical for astrocyte morphogenesis, *in vitro and in vivo*.

## MATERIALS & METHODS

### Mice

All mice and rats were used in accordance with the Institutional Animal Care and Use Committee (IACUC), under the guidance of the Duke Division of Laboratory Animal Resources (DLAR) with protocol numbers A147–17-06, A117–20-05, and A103–23-04. All animals were housed in 12-hour light/dark cycles. Timed-pregnant CD1 female mice (RRID: IMSR_CRL:022) were used for PALE and iBioID experiments, obtained from Charles River Laboratories. The NL2 floxed (B6;SJL-Nlgn2tm1.1Sud/J; Stock #025544, RRID:IMSR_JAX:025544), and Ai14 (RTM) (B6;129S6-Gt(ROSA)26Sortm14(CAG-tdTomato)Hze/J; Stock #007908, RRID:IMSR_JAX:007908) animals were obtained through Jackson Laboratory. CD Sprague-Dawley IGS Rats (SD-001, RRID: IMSR_CRL:400) were used for primary culture preps, obtained from Charles River Laboratories. Mice and rats of both sexes were pooled and included in all experiments.

### Plasmids:

#### shRNA plasmids:

shRNAs for NL1(RRID:Addgene_59339), 2 (RRID:Addgene_59358), and 3 (RRID:Addgene_59359) and scrambled NL shRNA control (shCtrl) constructs were described previously(Stogsdill et al., 2017).

shRNAs were cloned into pLKO.1 vector (a kind gift from David Root, Addgene plasmid #10878, RRID: Addgene_10878). A CAG-eGFP sequence was introduced into the vector for expression in primary astrocytes. Antisense shRNA sequences:

- Nedd4l: TTTATTAAGGGCTTTGCCTGC (Horizon Discovery, TRCN0000086868)

For PALE, the same shRNA sequence was cloned into a piggyBAC transposon system expressing mCherry CAAX and transfected with a GLAST-piggyBACase plasmid (a kind gift from Dr. Joseph LoTurco) as previously described(Tan et al., 2023). The Nedd4l shRNA in the piggybac backbone is available at Addgene (RRID:Addgene_249589).

#### NL chimeric plasmids:

Chimeric HA-NL2 constructs were a kind gift from Dr. Roger Nicoll and described previously(Nguyen et al., 2016) (NL2/1 Chimera RRID_Addgene_249601; NL2/3 Chimera RRID_249602). These constructs were modified to be resistant to shNL2(Stogsdill et al., 2017).

#### HA-NL2 mutation plasmids:

Deletion of the PDZ domain (RRID:Addgene_249594), poly-proline domain (RRID:Addgene_249595), and mutation of WW domains (PPDY → AAAA in NL2 (RRID:Addgene_249600), Large WW Deletion (a.a. 766–796 deletion, RRID:Addgene_249599), PPDY → AAAA in NL1/2 chimera (RRID:Addgene_249597) or LPDY → PPDY in NL3 chimera (RRID:Addgene_249598)) and K749R (RRID:Addgene_249588) were performed using custom gene blocks (IDT) in the HA-NL2-RM backbone. Mutation of the gephyrin binding residue in HA-NL2-RM (HA-NL2-GBM-RM RRID:Addgene_249596) was performed via site-directed mutagenesis using the QuikChange Lightning kit (Agilent) and the following primers:

Sense: 5’ GCGCCCTGCCTGTgcagccGACgctACCCTGGCCTTGCGCCG 3’

Antisense: 5’ GCCCGGCGCAAGGCCAGGGTAGCGTCggctgcACAGGCAG 3’.

#### TurboID plasmids:

pZac2.1-gfaABC1D-Turbo-BirA plasmid was a kind gift from Dr. Scott Soderling. The NL-Turbo fusion constructs were made by PCR amplification from the HA-tagged expression plasmids and subsequent In-Fusion cloning (Takara). A flexible glycine hexamer linker was inserted between the NL expression sequence and the Turbo-BirA. pZac2.1-GFABC1D-Turbo-BirA was digested with XhoI and NotI to release the Turbo-BirA to insert HA-NL1/2/3-Turbo-BirA. The gfaABC1D promoter was swapped for hSyn1 for neuronal expression of NL2-TurboID. The TurboID constructs are available through Addgene: gfaABC1D HA-NL2 Turbo RRID:Addgene_249590; gfaABC1D HA-NL1 Turbo RRID:Addgene_249591; gfaABC1D HA-NL3 Turbo RRID:Addgene_249592; hSyn1 HA-NL2 Turbo RRID:Addgene_249593.

#### Expression plasmids:

HA-NL1 (RRID:Addgene_15227), HA-NL2 (RRID:Addgene_15259) and HA-NL3 (RRID:Addgene_59318) were previously described(Stogsdill et al., 2017). V5-Nedd4l was made by custom geneblock cloning (IDT) from the HA-Nedd4l (human sequence, a kind gift from Joan Massague (Addgene plasmid # 27000; RRID:Addgene 27000)). GFP-Ubiquitin was a kind gift from Nico Dantuma (Addgene plasmid # 11928; RRID:Addgene 11928). For NL2 rescue PALE experiments, the CMV promoter was replaced with GfaABC1D(Lee et al., 2008) for both HA-NL2 (RRID:Addgene_249587) or HA-NL2K749R (RRID:Addgene_249588) via In Fusion Snap Assembly (Takara). pCAG-Cre was a gift from Connie Cepko (Addgene plasmid # 13775 ; http://n2t.net/addgene:13775 ; RRID:Addgene_13775).

#### CRISPR plasmids:

pX601-mCherry was a gift from Yuet Wai Kan (Addgene plasmid # 84039 ; http://n2t.net/addgene:84039 ; RRID:Addgene_84039). The CMV promoter was replaced with GfaABC1D promoter from the Turbo-BirA plasmids. SaCas9 gRNAs targeting rat *Nlgn2* were designed using CRISPOR(Concordet and Haeussler, 2018) software, and cloned into the BsaI sites in the pX601 vector.

- *Nlgn2* gRNA1: AAGAACTGCACGACCGGGCC
- *Nlgn2* gRNA2: GTGGTGAACACAGCCTACGG (RRID:Addgene_250933)
- *Nlgn2* gRNA3: GCCACACTCAACTACCGTCT (RRID:Addgene_250934)

For the CRISPR GFP translation assay, we cloned the NL2 gRNAs and their PAM sequences upstream of and in frame with GFP under the control of the chicken beta actin promoter.

### Rat astrocyte and neuron co-culture:

Astrocyte and neuron cultures were performed as described(Baldwin et al., 2021; Stogsdill et al., 2017). Briefly, P1 rat cortices were dissected and digested in ~7.5 units/ml Papain (Worthington). A single-cell suspension was extracted by gentle pipetting with ovomucoid solutions (Worthington). Cells were filtered through a 20μm mesh membrane (Elko Filtering 03–20/14) and then split for astrocyte and neuron cultures.

For neuronal isolation, cortical cells were applied to negative panning dishes coated with Bandeiraea Simplicifolia Lectin 1 (x2), followed by AffiniPure goat anti-mouse IgG+IgM (H+L) (Jackson ImmunoResearch 115–005-044, RRID: AB_2338451), and AffiniPure goat anti-rat IgG+IgM (H+L) (Jackson ImmunoResearch 112–005-044, RRID:AB_2338094) antibodies to bind unwanted cells and debris. Cell suspensions were passed onto positive panning dishes coated with mouse anti-L1 (ASCS4, Developmental Studies Hybridoma Bank, Univ. Iowa, RRID:AB_528349) to bind cortical neurons (>95% neuron). Adherent cells were collected following several washes with DPBS (Gibco 14287) (supplemented with BSA and insulin) and forceful pipetting with a P1000 pipette. Isolated neurons were pelleted (11 minutes at 200 g) and resuspended in serum-free neuron growth media (NGM; Neurobasal, B27 supplement, 2 mM L-Glutamine, 100 U/mL Pen/Strep, 1 mM NaPyruvate, 4.2 μg/mL Forskolin, 50 ng/mL, BDNF, and 10 ng/mL CNTF). 100,000 neurons were plated onto 12 mm glass coverslips (24-well plate) coated with 10 μg/mL poly-D-lysine (PDL, Sigma P6407) and 2 μg/mL laminin, and incubated at 37oC in 10% CO2. On DIV2, neurons were treated with 10μM AraC for one day. Further, on DIV2, media was changed to Neurobasal Plus Growth Media (NGM+; Neurobasal Plus, B27 Plus supplement, 100 U/mL Pen/Strep, 1 mM NaPyruvate, 4.2 μg/mL Forskolin, 50 ng/mL, BDNF, and 10 ng/mL CNTF). Neurons were given a 50% media change every third day until astrocyte co-culture. A detailed protocol can be found at: https://www.protocols.io/view/glia-free-cortical-neuron-culture-36wgq3r35lk5/v1.

For astrocyte cultures, cortical cells were centrifuged and resuspended in astrocyte growth medium (AGM; DMEM (Gibco 11960), 10% FBS, 10 μM, hydrocortisone, 100 U/mL Pen/Strep, 2 mM L-Glutamine, 5 μg/ml Insulin, 1 mM NaPyruvate, 5 μg/ml N-Acetyl-L-cysteine). On the third day in vitro (DIV), astrocytes were washed with DPBS and then shaken vigorously by hand until only adherent astrocytes remained. DPBS was replaced with fresh AGM. AraC was added to the culture at DIV5 for two days. On DIV7, astrocytes were passaged via Trypsin (Gibco) into 6- or 12-well dishes and then transfected on DIV8 with Lipofectamine-LTX. On DIV10, transfected astrocytes were trypsinized and resuspended into NGM+ and plated on neurons at a density of 20,000 cells per well (24 well plates). Cells were fixed after 48 hours of co-culture with warm 4% PFA (Electron Microscopy Sciences) for exactly 7 minutes.

### Immunocytochemistry:

After fixation in 4% warmed PFA for 7 minutes (except in the case of surface staining in which cold PFA was used for 5 minutes), coverslips were blocked in a blocking buffer (150 NaCl, 50 Tris-base, 1% BSA, 100 mM L-lysine, 0.04% Sodium azide, pH 7.4) containing 0.2% Triton X-100 (omitted in surface staining) and 50% NGS was added to the coverslips for 30 minutes, followed by 2 washes of DPBS. Primary antibodies were diluted in a blocking buffer containing 10% NGS and added to the coverslips overnight at 4C. The following antibodies were used: GFAP (1:4000, rabbit, DAKO Z0334, RRID:AB_10013382), GFP (1:1000, chicken, Millipore AB16901, RRID:AB_90890), HA (1:1000, rat, Roche 11867423001, RRID:AB_390918), RFP (1:1000, rabbit, Rockland 600–401-379, RRID:AB_2209751). The next day, coverslips were washed 3 times with PBS, and Alexa Fluor conjugated secondaries (Invitrogen: Rb 594, RRID:AB_2534079; Ck 488 RRID:AB_2534096; Rat 594 RRID:AB_10561522) were diluted 1:1000 in blocking buffer containing 10% NGS and incubated for 2 hours at room temperature.

After the incubation, the coverslips were washed 3 times and mounted onto glass slides (VWR) with Vectashield mounting media containing DAPI (Vector Laboratories). Imaging was described as before(Stogsdill et al., 2017) on an AxioImager M1 (Zeiss) and a Keyence BZ-X800 at 40X magnification by a blinded investigator. Images were analyzed with Sholl analysis in FIJI (NIH).

### CRISPR genome editing efficiency:

To quantify the percent of frameshift inducing mutations, we cloned all 3 NL2 gRNAs and their respective PAM sequences upstream of and in frame with a GFP coding sequence. In HEK293T (ATCC, RRID:CVCL_4V93) cells we co-transfected the pX601-CMV-SaCas9-U6 gRNA plasmids (containing each NL2 gRNA) with the GFP target plasmid. Three days after transfection, the cells were harvested in 1X MPER (Thermo Scientific), incubated at 4C with end-over-end rotation for 10 minutes and then spun at 15,000 × g for 5 minutes. The supernatant was kept for downstream western blot analysis. GFP expression on the resulting western blot was quantified using ImageJ and normalized to GAPDH expression. In this assay, a reduction of GFP expression is interpreted to be genomic cutting by the corresponding gRNA.

### In vitro Sholl Analysis:

Sholl Analysis was performed in FIJI (NIH, v1.53c) using the Sholl Analysis plug-in (v4.0.1). The number of intersections is measured with concentric circles at every 1μm starting from a radius of 10μm from the center of the nucleus. Resulting values were then analyzed in R (v4.0.0 and above) using an in-home pipeline which can be found here: https://github.com/Eroglu-Lab/In-Vitro-Sholl. The final graphs are the average of each biological replicate, not of each image. The line graphs were generated using the LOESS method (ggplot2 [Hadley Wickham], stat_smooth function) and the variance (standard error) is represented with the colored lines.

### Postnatal astrocyte labeling by electroporation (PALE):

PALE was performed as previously described(Baldwin et al., 2021). Briefly, P0-P1 pups were placed on a glove on ice for < 5 minutes until unresponsive. 1–2μg (in 1–2μl total volume) of plasmid was loaded into a pulled glass pipette containing 1% fast green dye (Sigma F7252). The glass pipette was then inserted into one lateral ventricle of the mouse brain and DNA was injected. Electrodes were then positioned above the visual cortex (positive end) and below the jaw (negative end) and five 50ms pulses of 100V with an interpulse interval of 950ms were applied. Pups recovered on a heating pad and were then returned to the home cage with the dam. Each litter received a random assignment of experimental groups. All animals that recovered after surgery with no visible health issues were kept for the analysis. Animals were transcardially perfused with 4% PFA at P21.

### Immunohistochemistry:

Mice were perfused transcardially with 4% PFA and were transferred to 4% PFA overnight before incubation with 30% sucrose in TBS. Coronal sections were collected on a cryostat (Leica) at either 40μm (iBioID, synaptic staining) or 100μm (territory volume and 3D Sholl). Sections were washed 3 times for 10 minutes on a shaker in 0.2%(40μm sections) or 0.4%(100μm sections) Triton-X in TBS (TBST). Sections were blocked in 10% Goat serum in TBST for 1 hour on the shaker at room temperature.

Primary antibodies: PSD95 (1:300, rabbit, Invitrogen 51–6900, RRID:AB_2533914), Gephyrin (1:500, mouse, Synaptic Systems 147011, RRID:AB_2810215), RFP (1:1000, chicken, Rockland 600–901-379, RRID:AB_10704808, used for synaptic staining), Bassoon (1:500, guinea pig, Synaptic Systems 141318, RRID:AB_2927388), HA (1:200, rat, Roche 11867423001, RRID:AB_390918), RFP [for detecting mCherry CAAX in shRNA PALE experiments] (1:1000, rabbit, Rockland 600–401-379, RRID:AB_2209751), RFP nanobody 568 coupled [for detecting tdTomato in Ai14 mice for NL2 rescue PALE experiments] (1:100, Synaptic Systems,N0404-AF568-L, RRID:AB_2209751), Sox9 (1:1000, Rabbit, Millipore AB5535, RRID:AB_2239761), NeuN (1:1000, Mouse, Millipore MAB377, RRID:AB_2298772) were diluted in fresh blocking buffer and incubated either overnight (40μm) or for 3 nights (100μm). Alexa Fluor or Streptavidin conjugated fluorescent secondaries (Invitrogen: Rabbit 405+ RRID:AB_2890548; Mouse IgG1 488 RRID:AB_2535764, Chicken 568 RRID:AB_2534098, Guinea Pig 647 RRID:AB_2535867, Rat 488 RRID:AB_2534074, Rabbit 594 RRID:AB_2534095, Streptavidin Alexa Fluor conjugate (Thermo Fisher, S32357)) were diluted in blocking buffer at 1:200 and incubated on tissue for 3 hours at room temperature. DAPI was added in the final wash before mounting. Sections were mounted on glass slides with mounting media (90% Glycerol, 20 mM Tris pH 8.0, 0.5% n-Propyl gallate).

### Synapse quantification:

Images for synapse quantification were acquired on an Olympus FV 3000 at 60X magnification with 1.64X zoom, in 1μm stacks with a 0.34μm step size. We used SynBot(Savage et al., 2024) (https://github.com/Eroglu-Lab/Syn_Bot) in FIJI to quantify the colocalization of Bassoon and Psd95 (excitatory) or Bassoon and Gepyhrin (inhibitory) synapses for the entire image using manual thresholding. Using a custom macro in FIJI (https://github.com/Eroglu-Lab/Sakers-et-al-2025), we then cropped the binarized synaptic output images to draw an ROI around the labeled astrocyte and quantify the number of synaptic puncta within and outside the ROI. These synaptic puncta were normalized to the corresponding ROI to obtain a synapse density. The data were analyzed with a linear mixed effects ANOVA in R.

### Imaris territory volume and 3D Sholl analysis:

100μm sections were imaged on an Olympus FV 3000 at 60X magnification, with 2X zoom. Single astrocytes were captured in a frame in a Z stack at 0.5μm step size to capture the whole astrocyte. Imaris v9.9 (Bitplane) was used to reconstruct images using the filament tracer in 3D. We used two custom extensions to quantify filament complexity (3D Sholl analysis) every 5μm from the cell nucleus [note that this is different from 2D Sholl analysis where a measurement is taken every 1μm from the cell nucleus]. A linear mixed model followed by Dunnett’s post-hoc test was used to analyze the 3D Sholl data using a custom R script found at: https://github.com/Eroglu-Lab/In-vivo-Sholl-Analysis. The whole astrocyte territory volume was calculated using the Convex Hull function in Imaris on the cell surface territory. Analysis of territory volume was analyzed in R using a one-way repeated measures ANOVA to account for multiple images taken per animal, followed by Dunnett’s post-hoc test.

### Adeno-associated virus production:

Purified AAV was produced as described previously(Baldwin et al., 2021). Briefly, HEK293T cells were transfected with pAd-DELTA F6, serotype plasmid AAV PHP.eB, and AAV plasmids (pZac2.1 Turbo ID constructs). Three days after transfection, cells were collected in 15 mM NaCl, 5 mM Tris-HCl, pH 8.5, and lysed with repeat freeze-thaw cycles followed by treatment with Benzonase (Novagen 70664) at 37°C for 30 minutes. Lysed cells were pelleted by centrifugation and the supernatant, containing AAV was applied to an Optiprep density gradient (Sigma D1556, 15%, 25%, 40%, and 60%) and centrifuged at 67,000 rpm using a Beckman Ti-70 rotor for 1 hour. The AAV-enriched fraction was isolated from between 40% and 60% iodixanol solution and concentrated by repeated washes with sterile PBS in an Amicon Ultra-15 filtration unit (NMWL: 100 kDa, Millipore UFC910008) to a final volume of ~100 μL and aliquoted for storage at −80°C.

### In vivo BioID:

Pregnant CD1 dams were ordered from Charles River Laboratories. On P1, pups were anesthetized by hypothermia and subsequently injected intracranially at a depth of 2mm with 1μl equal viral titers of either NL2-Turbo-BirA or Turbo-BirA as previously described. Mice were treated with 0.75mL of 5mM Biotin in sterile PBS via subcutaneous injections once a day from P18 to P20. On P21, mice were sacrificed with 200 mg/kg tribromoethanol (avertin) and transcardially perfused with TBS/Heparin for 5 minutes to wash out excess biotin. Subsequently, cortices were rapidly dissected in DPBS and flash-frozen in liquid nitrogen.

All solutions were made fresh using proteomics-grade chemicals and solvents. Brains were homogenized with approximately 12 strokes in a glass homogenizer and Teflon pestle in 1mL Lysis buffer [50mM Tris/HCl pH7.5, 150mM NaCl, 1mM EDTA with protease inhibitors (Roche)] on ice. An equal volume of 2X RIPA [50mM Tris/HCl pH7.5, 150mM NaCl, 1mM EDTA, 0.4% SDS, 2%TritonX100, 2% deoxycholate] was added to the lysates. Lysates were then sonicated (level 9) for 30 seconds (3 × 10s) total, followed by centrifugation at 15,000 RPM for 10 minutes at 4C. The supernatant was transferred to an ultracentrifuge 1.5mL tube (Beckman Coulter) and spun in a TLA-55 rotor (Beckman coulter) in an ultracentrifuge at 40,000 RPM for 30 minutes at 4C. Supernatants were transferred to a low-protein binding tube (Eppendorf) and SDS was added to a final concentration of 1%. Samples were then briefly vortexed and heated at 45C for 45 mins, cooled on ice, and spun at 15,000RPM for 30 minutes at 4C. 70 μl Neutravidin beads (ThermoFisher 29202) were prepared per condition by washing with 1mL of 2X RIPA two times. The samples were transferred to 15mL tubes (1 brain per IP, 3 brains per condition), and washed Neutravidin beads were added to the sample supernatant. 30ul of the supernatant was saved for western blot analysis. The beads and samples were incubated overnight at 4C with rotation. The next day beads were sequentially washed with 1mL of the following solutions for 10 minutes with rotation, followed by two-minute spins: 2% SDS x2, Wash buffer (1% TritonX100, 1% deoxycholate, 25 mM LiCl) x2, 1M NaCl x2, 50mM Ammonium bicarbonate x5. Elution buffer was prepared during the last washes by adding 1.2mg of biotin to 2X sample buffer [4% SDS, 20% glycerol, 0.1% beta-mercaptoethanol, 125mM Tris pH 6.8 to 20mL with keratin-free water]. 70ul of elution buffer was added to the beads and samples were then heated at 60C for 15 minutes to elute bound proteins. The sample was stored at −80C before mass spec analysis, and 5ul of the eluate was saved for western blotting. All washes and elution were done in a tissue culture hood, in sterile conditions to avoid keratin contamination.

### Mass spectrometry:

Sample Preparation: The Duke Proteomics Core Facility (DPCF) received 6 samples (3 replicates each of two conditions). Samples were reduced with 10 mM dithiothreitol for 30 min at 80C and alkylated with 20 mM iodoacetamide for 30 min at room temperature. Next, they were supplemented with a final concentration of 1.2% phosphoric acid and 597 μL of S-Trap (Protifi) binding buffer (90% MeOH/100mM TEAB). Proteins were trapped on the S-Trap, digested using 20 ng/μl sequencing grade trypsin (Promega) for 1 hr at 47C, and eluted using 50 mM TEAB, followed by 0.2% FA, and lastly using 50% ACN/0.2% FA. All samples were then lyophilized to dryness and resuspended in 12μL 1%TFA/2% acetonitrile containing 12.5 fmol/μL yeast alcohol dehydrogenase (ADH_YEAST). A QC Pool was created from 3uL of each sample. All QC Pools were run periodically throughout the acquisition period.

Quantitative Analysis, Methods: Quantitative LC/MS/MS was performed on 3 μL of each sample, using a nanoAcquity UPLC system (Waters Corp) coupled to a Thermo Orbitrap Fusion Lumos high-resolution accurate mass tandem mass spectrometer (Thermo) via a nanoelectrospray ionization source. Briefly, the sample was first trapped on a Symmetry C18 20 mm × 180 μm trapping column (5 μl/min at 99.9/0.1 v/v water/acetonitrile), after which the analytical separation was performed using a 1.8 μm Acquity HSS T3 C18 75 μm × 250 mm column (Waters Corp.) with a 90-min linear gradient of 5 to 30% acetonitrile with 0.1% formic acid at a flow rate of 400 nanoliters/minute (nL/min) with a column temperature of 55C. Data collection on the Fusion Lumos mass spectrometer was performed in a data-dependent acquisition (DDA) mode of acquisition with a r=120,000 (@ m/z 200) full MS scan from m/z 375 – 1500 with a target AGC value of 2e5 ions. MS/MS scans were acquired at a Rapid scan rate (Ion Trap) with an AGC target of 5e3 ions and a max injection time of 100 ms. The total cycle time for MS and MS/MS scans was 2 sec. A 20s dynamic exclusion was employed to increase the depth of coverage. The total analysis cycle time for each sample injection was approximately 2 hours. Following 9 total UPLC-MS/MS analyses (excluding conditioning runs, but including 3 replicate QC Pool), data was imported into Proteome Discoverer 2.3 (Thermo Scientific Inc.), and analyses were aligned based on the accurate mass and retention time of detected ions (“features”) using Minora Feature Detector algorithm in Proteome Discoverer. Relative peptide abundance was calculated based on the area-under-the-curve (AUC) of the selected ion chromatograms of the aligned features across all runs. The MS/MS data was searched against the SwissProt M. musculus database (downloaded in Apr2017) and an equal number of reversed-sequence “decoys” for false discovery rate determination. Mascot Distiller and Mascot Server (v 2.5, Matrix Sciences) were utilized to produce fragment ion spectra and to perform the database searches. Database search parameters included fixed modifications on Cys (carbamidomethyl) and variable modifications on Meth (oxidation) and Asn and Gln (deamidation). Peptide Validator and Protein FDR Validator nodes in Proteome Discoverer were used to annotate the data at a maximum 1% protein false discovery rate.

#### Statistical analysis of iBioID data:

In vivo BioID has an inherently high background in mass spectrometry due to the time necessary for biotin to accumulate in the system, and the diffuse nature of AMP-biotin. We controlled for this background using a soluble (non-bait) TurboID enzyme in both astrocytes and neurons, as this would accurately replicate the high background in bait conditions. Further, to account for differences in expression due to the size differenced of soluble TurboID and NL-tagged TurboID, we employed a robust-mean normalization which excludes the top and bottom 10% of signal intensities and mitigates the influences of outliers. The BioID datasets were generated from distinct viral constructs and cellular contexts, thus necessitating an enrichment-over-control design. To do so, we calculated the fold changes and used a two-tailed heteroscedastic t-test on log2-transformed data comparing the NL2 and Turbo BioID groups, and performed FDR correction to obtain high-confidence putative interactors.

### Western blotting:

iBioID: 5ul of IP eluate and 30ul of sonicated lysate were run on a 4–15% Mini-Protean precast gel (BioRad). After transfer, membranes were blocked in Odyssey blocking buffer (LI-COR) diluted in PBS (1:1) followed by incubation in the same buffer with Alexa 680 conjugated streptavidin (Invitrogen). Membranes were imaged on a LI-COR Odyssey CLx.

All other blots: Equal protein (measured by Bicinchoninic Acid Protein Assay (BCA, Pierce)) was loaded on a 4–15% Mini-Protean precast gel (BioRad). In the case of immunoprecipitations, input samples were loaded as an equal percentage of total sample homogenate, and the entirety of the IP eluate was run per lane. Gels were transferred using a Power Blotter System (Invitrogen) and then blocked in a Blocking Buffer for Fluorescent Western Blotting (Rockland, MB-070) diluting in PBS (1:1). The following primary antibodies were used for blotting: Rabbit anti-Nedd4l (Cell Signaling Technology, 4013S, 1:1000, RRID:AB_1904063), Rabbit anti-V5 (Cell Signaling Technology, 13202S, 1:500, RRID:AB_2687461), Chicken anti-HA (Aves Labs, ET-HA100, 1:2000, RRID:AB_2313511), Rabbit anti-GFP (Novus Biologicals, NB600–308, 1:1000, RRID:AB_10003058), Mouse anti-Gapdh (Abcam, ab8245, 1:2500, RRID:AB_2107448), Mouse anti-Actin (Sigma-Aldrich, A5441, 1:2000, RRID:AB_476744) diluted in blocking buffer. LI-COR 680 or 800 fluorescent secondary antibodies (Chicken 800 RRID:AB_10974977; Rabbit 800 RRID:AB_621848; Mouse 800 RRID:AB_621842, Rabbit 680 RRID:AB_621845, Chicken 680 RRID:AB_1850018) were diluted 1:2500 in blocking buffer and blots were imaged on a LI-COR Odyssey CLx.

### Lentivirus production and transduction:

8–10 million 293T cells were plated in a T-75 culture flask and transfected using Xtremegene HP Transfection Reagent (Roche, 06366236001) with the following plasmids: pLKO.1 puromycin-resistant shRNA plasmids (described above), VSVG (an envelope plasmid), and dR8.91 packaging plasmid. One day post-transfection, the media was replaced with astrocyte growth media, and lentivirus was collected on days 2 and 3 and filtered through a 40μm syringe filter. Media was tested for lentiviral particles using Lenti-X Go Stix (Takara, 631280). Astrocytes were treated with 500μl of lentiviral media supernatant, and 1μg/mL polybrene on DIV8. On DIV10, astrocytes were treated with 1μg/mL puromycin every other day until a non-transduced well was completely dead. Cells were lysed for qPCR as described below. Cells collected for western blot were collected in T-PER (Thermo Scientific, 78510) and protein was measured using a BCA protein assay (Thermo Scientific, 23227).

### Gene ontology analysis:

Gene ontology analysis was performed in R using the package clusterProfiler v4.6.2(Wu et al., 2021). Proteins from iBioID lists that were at least 1.5 fold enriched at a p-value of <0.05, compared to respective controls described in legends, were subjected to Biological Processes enrichment. Resulting output was reduced using the simplify function in clusterProfiler. The top 5–10 categories (sorted by adjusted p-value) were plotted in R. The entire enrichment can be found in Supplemental Dataset 2.

### Immunoprecipitation:

4 million 293T cells (ATCC, RRID:CVCL_4V93) (passage #8–15) were plated in 10cm dishes and then transfected the next day with 2.5μg of each plasmid using the Xtremegene HP Transfection Reagent (Roche, 06366236001) according to the manufacturer’s protocol. 48 hours post transfection cells were washed with 1X TBS supplemented with calcium and magnesium and pelleted. Cell pellets were resuspended in 400μl of lysis buffer (25mM Tris pH 7.4, 1mM Calcium Chloride, 1mM Magnesium Chloride, 150mM Sodium Chloride, 0.5% NP-40, 1X EDTA-free protease inhibitor cocktail (Roche)) and centrifuged at 20,000×g for 10 minutes. 5% of the supernatant was saved for input samples, and the remaining lysate was diluted with lysis buffer without NP-40 for IP. Lysate was added to 30μl of magnetic anti-HA beads (Pierce, 88836) overnight at 4C with rotation. Beads were collected on a magnetic rack and washed 3 times with wash buffer (25mM Tris pH 7.4, 1mM Calcium Chloride, 1mM Magnesium Chloride, 150mM Sodium Chloride, 0.1% NP-40, 1X EDTA-free protease inhibitor cocktail (Roche)). Beads were eluted with 2X Laemmli buffer (Bio-Rad, 1610737) at 42C for 15 minutes and then denatured with 5% β-mercaptoethanol at 45C for 45 minutes before loading on SDS-PAGE.

### Ubiquitination assay:

293T cells were transfected as indicated in the immunoprecipitation subsection. 48 hours post-transfection, cells were treated with 25μM MG-132 (Sigma-Aldrich, 474790) for 2–4 hours to block the proteasome. Cells were washed 3 times in ice-cold 1X TBS supplemented with magnesium, calcium, and N-ethylmaleimide (NEM) (Sigma-Aldrich, E3876) to block deubiquitinases and then pelleted. Cells were resuspended in 1X RIPA buffer with SDS (50 mM Tris-HCl pH 7.4, 150 mM NaCl, 1% NP-40, 0.5% Sodium Deoxycholate, 1% Sodium dodecyl sulfate, supplemented with 1X protease inhibitor and 20mM NEM) and then boiled for 5 minutes to disrupt protein-protein interactions. The lysates were diluted 10X with 1X RIPA without SDS and incubated on a rotator at 4C for 1 hour. The lysates were then pelleted at 15,000×g for 15 minutes and the supernatant was added to magnetic anti-HA beads, and 5% of the supernatant was collected for input fraction. Elution was performed identically to the immunoprecipitation subsection above. For ubiquitination assay in primary rat astrocytes, 5 million DIV7 astrocytes (prepared as described above) were nucleofected with the Basic Glial Cells Nucleofection Kit (Lonza) and 5ug of each plasmid. 24 hours after nucleofection, the media was changed to remove dead cells and nucleofection solution. Ubiquitination IP was performed 3 days after nucleofection in the same manner as 293T cells.

### Degradation assay:

350,000 HEK 293T cells were plated into wells of a 6-well plate and then transfected one day later with 1μg of each plasmid via Xtremegene HP Transfection Reagent reagent (Roche #06366236001). 48 hours later cells were treated with cycloheximide (100μg/mL). At each time point, the collected cells were washed with 1X TBS with magnesium and calcium, pelleted, and then resuspended in 1X RIPA (Sigma-Aldrich) supplemented with protease inhibitors. Lysates were incubated at 4C with rotation for 40 minutes, centrifuged at 20,000×g at 4C and supernatants were saved for protein quantitation via BCA. 5μg of total protein was loaded on a 4–15% SDS-PAGE. Three independent cultures were used for statistical analysis.

### Statistics:

Experiments were randomized where applicable and analyses were run by an experimenter blind to the condition. All in vitro experiments were performed at least 3 times (3 different cultures), with 20+ cells imaged per replicate. Note that some conditions were plotted across several figures (i.e. shNL2 in Figure S1, Figure 1; shNL2 + HA-NL2 Figure 1D and F and S1, and shNL2 in Figure 5), as experiments were done in parallel. All analyses were done in R (v4.0.0 and above). An R script with reproducible data can be found at the following links for both in vitro analysis (https://github.com/Eroglu-Lab/In-Vitro-Sholl) and in vivo analysis (https://github.com/Eroglu-Lab/In-vivo-Sholl-Analysis). Statistical analyses were performed using N of independent culture or animal rather than image to avoid pseudoreplication artifacts. Specific details on sample size, and test applied can be found in the figure legends and Methods subsections and in Table S1.

## Supplementary Material

Supplement 1

Supplement 2

Supplement 3

Supplement 4

## Data Availability

The mass spectrometry proteomics data have been deposited to the ProteomeXchange Consortium via the PRIDE(Perez-Riverol et al., 2022) partner repository with the dataset identifier PXD049185. All data in this manuscript can be found in the attached Source Data file.
