# Supplementary material for: Neuroligin-2 is ubiquitinated by Nedd4l to control developmental astrocyte morphogenesis
Source: bioRxiv. 2025 Dec 16:2025.12.15.694023. Preprint. [Version 2] doi: 10.64898/2025.12.15.694023 (PMC12724466; doi:10.64898/2025.12.15.694023)
Supplement: Supplement 3 [file media-3.pdf]

Figure Panel	Description	Statistical Analysis
1D	Sholl analysis	Linear mixed effects model (LME) ANOVA F(3,12) = 9.074, p=0.0021. Dunnett's posthoc tests values on graph [control condition is shNL2+HA-NL2-RM]. N = 48-96 cells across conditions, 3 biological replicates
1F	Sholl analysis	Linear mixed effects model (LME) ANOVA F(3, 11) = 3.15, p=0.0686. Dunnett's posthoc tests values on graph [control condition is shCtrl+HA-NL2-RM]. N = 53-97 cells across conditions, 3 biological replicates
2C	Sholl analysis	Linear mixed effects model (LME) ANOVA F(3, 8) = 0.32, p=0.81. Dunnett's posthoc tests values on graph [control condition is shNL2+HA-NL2-RM]. N = 36-46 cells across conditions, 3 biological replicates
2F	Sholl analysis	Linear mixed effects model (LME) ANOVA F(3, 12) = 8.8, p=0.0023. Dunnett's posthoc tests values on graph [control condition is shNL2+HA-NL2-RM]. N = 57-97 cells across conditions, 4 biological replicates
2J	Sholl analysis	Linear mixed effects model (LME) ANOVA F(3, 13) = 9.9, p=0.0011. Dunnett's posthoc tests values on graph [control condition is shNL2+HA-NL2-RM]. N = 71-84 cells across conditions, 4 biological replicates
4A	Overlap of BioID candidates with previously published datasets	Fisher's exact test. Exact p-values:<br>Kang et al [Astrocyte NL2 BioID] = 1.4e-40

		Kang et al [Neuron NL2 BioID] = 3.5e-30<br>Loh et al [Astrocyte NL2 BioID] = 4.4e-6<br>Loh et al [Neuron NL2 BioID] = 7.1e-18<br>Poulopoulos et al [Astrocyte NL2 BioID] = 1.4e-13<br>Poulopoulos et al [Neuron NL2 BioID] = 3.9e-13
4B	BioID enrichment	Heteroscedastic t-test. P-values printed on graph are uncorrected p-values
4C	Gene ontology [Astrocyte NL BioID comparison]	Over-representation analysis with FDR correction. Used clusterProfiler package (v4.16.0) compareCluster function.
4D	Gene ontology	Over-representation analysis with FDR correction. Used clusterProfiler package (v4.16.0) compareCluster function.
4E	BioID enrichment	Heteroscedastic t-test. P-values printed on graph are uncorrected p-values. Fold change is relative to cell-type specific soluble BirA control. Values come from Table S1.
5B	Sholl Analysis	Linear mixed effects model (LME) ANOVA F(3, 13) = 38.104, p=1.05e-6. Tukey's posthoc tests values on graph [control condition is shCtrl]. N = 60-100 cells across conditions, 4 biological replicates
5E	Territory volume analysis	Nested ANOVA F(1,8) = 11.95, p=0.0023. 4-7 animals imaged per condition, with at least 2 images per animal acquired.
5F	Sholl analysis	Linear mixed effects model (LME) ANOVA F(1, 9) = 5.59, p=0.04. 4-7 animals

		imaged per condition, with at least 2 images per animal acquired.
6C	Densitometry quantification of I.P. bands	Paired student's t-test. P=0.103. N=4 independent replicates per condition
6E	Densitometry quantification of I.P. bands	Paired student's t-test. P=0.034. N=3 independent replicates per condition
6G	Densitometry quantification of I.P. bands	Paired student's t-test. P=0.043. N=3 independent replicates per condition
6I	Densitometry quantification of I.P. bands	Paired student's t-test. P=0.006. N=3 independent replicates per condition
7B	Quantification of high molecular weight HA-NL2 [> 100kDa]	Paired student's t-test. P=0.04. N=3 independent replicates per condition
7D	Densitometry quantification of HA-NL2 western blots	Two-way ANOVA: Condition: $F(1,16) = 15.018$, $p=0.00134$ Timepoint: $F(3,16) = 0.41$, $p=0.748$ Interaction Condition:Timepoint: $F(3, 16) = 2.242$, $p=0.123$
7F	Densitometry quantification of HA-NL2 western blots	Two-way ANOVA: Condition: $F(1,16) = 1.392$, $p=0.255$ Timepoint: $F(3,16) = 1.681$, $p=0.211$ Interaction Condition:Timepoint: $F(3, 16) = 2.328$, $p=0.113$
8B	Sholl analysis	Linear mixed effects model (LME) ANOVA $F(3, 8) = 13.49$, $p=0.0017$. Dunnett's posthoc tests values on graph [control condition is shCtrl]. N = 62-80 cells across conditions, 3 biological replicates
8D	Territory volume	Kruskal-Wallis rank sum test. $\chi 2 = 19.5$, df=3, $p=0.00013$. Wilcoxon rank sum post-hoc tests with Holm-Šídák multiple comparison correction are on the graph.

8E	Sholl analysis	Linear mixed effects model (LME) ANOVA F(3, 105) = 5.46, p=0.0016. Tukey's posthoc results in figure. N = 21-33 cells total from 6-7 animals per group, with at least 2 cells imaged per animals.
S1B	Sholl analysis	Linear mixed effects model (LME) ANOVA F(3, 10) = 12.145, p=0.0011. Dunnett's posthoc tests values on graph [control condition is shCtrl]. N = 53-96 cells across conditions, 3 biological replicates
S1F	Sholl analysis	Linear mixed effects model (LME) ANOVA F(2, 6) = 21.12, p=0.0019. Tukey's posthoc tests values on graph [control condition is shCtrl]. N = 40 cells across conditions, 3 biological replicates
S3C	Sholl analysis	Linear mixed effects model (LME) ANOVA F(1, 3) = 11.01, p=1.4E-6. Dunnett's posthoc tests values on graph [control condition is shNL2+HA-NL2-RM]. N = 14-56 cells across conditions, 3 biological replicates
S4A-F	BioID enrichment.	Heteroscedastic t-test. P-values printed on graph are uncorrected p-values. Fold change is relative to cell-type specific soluble BirA control. Values come from Table S1.
S5A	Densitometry of Nedd4l western blot, normalized to Actin	Student's t-test. P-value = 0.0004. N=3 independent replicates.
S6D	Quantification of excitatory and inhibitory synapse density in shNedd4l or shCtrl astrocytes	Linear mixed effects ANOVA.<br>Excitatory synapses: F(1,13) = 0.12, p=0.73<br>Inhibitory synapses: F(1,13) = 0.61, p=0.45

		N = 8 shCtrl animals, and 7 shNedd4l animals. 4-8 images per animal were acquired.
