# Supplementary material for: Neuroligin-2 is ubiquitinated by Nedd4l to control developmental astrocyte morphogenesis
Source: bioRxiv. 2025 Dec 16:2025.12.15.694023. Preprint. [Version 2] doi: 10.64898/2025.12.15.694023 (PMC12724466; doi:10.64898/2025.12.15.694023)
Supplement: Supplement 4 [file NIHPP2025.12.15.694023v2-supplement-4.pdf]

bioRxiv preprint doi: https://doi.org/10.64898/2025.12.15.694023; this version posted December 16, 2025. The copyright holder for this preprint (which was not certified by peer review) is the author/funder, who has granted bioRxiv a license to display the preprint in perpetuity. It is made available under aCC-BY 4.0 International license.

Supplemental Figure 1:

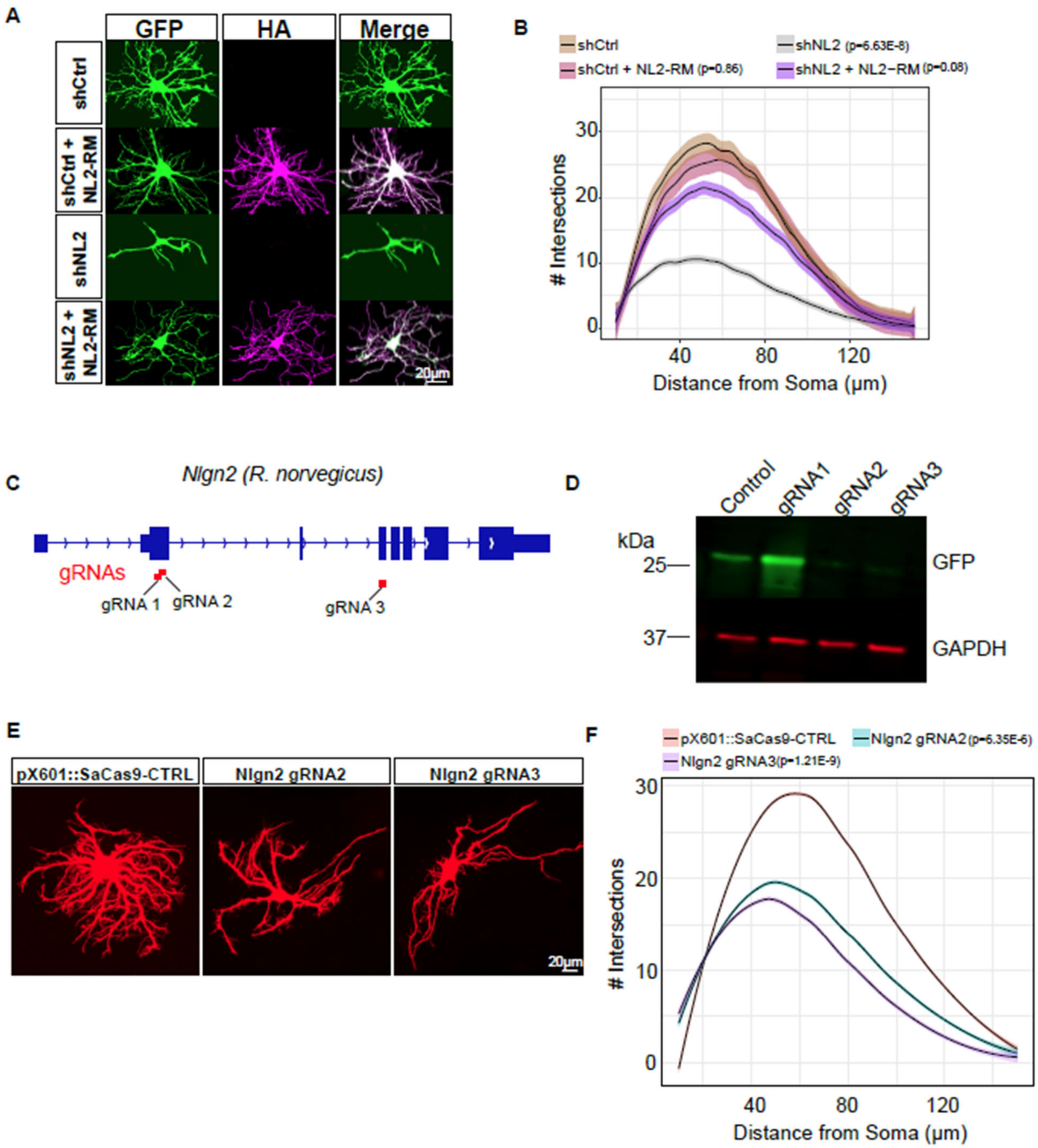

A) Representative images of astrocytes cultured on neurons. Astrocytes were transfected with either shCtrl or shNL2, alone or with HA-NL2-RM (Rescue mutant).

bioRxiv preprint doi: https://doi.org/10.64898/2025.12.15.694023; this version posted December 16, 2025. The copyright holder for this preprint (which was not certified by peer review) is the author/funder, who has granted bioRxiv a license to display the preprint in perpetuity. It is made available under aCC-BY 4.0 International license.

B) Sholl analysis of images represented in A. Linear-mixed model followed by ANOVA reveals the main effect of condition $F(3, 10)=12.145$, $p=0.0011$. P-values represent post hoc Dunnett's comparison to shCtrl. N = 53-96 cells per condition, across 3 biological replicates (independent culture experiments).

C) *Nlgn2* sequence illustrating location of the gRNAs using in D and E.

D) Western blot of GFP translation assay. The target sequence of the gRNAs is cloned upstream and in-frame with GFP. gRNAs are co-expressed and then lysates are collected and run in equal protein load on an SDS-PAGE. The western blot was probed for GFP and GAPDH, for normalization.

E) Representative images of astrocytes cultured on neurons. Astrocytes were transfected with pX601 (U6 gRNA GfaABC1D Cas9 T2A mCherry) prior to culturing on neurons.

F) Sholl analysis of images represented in C. Linear-mixed model followed by ANOVA reveals the main effect of condition $F(2, 6) = 21.12$, $p=0.0019$. P-values represent post hoc Tukey's comparison to shCtrl. N = 40 cells per condition, across 3 biological replicates (independent culture experiments).

bioRxiv preprint doi: https://doi.org/10.64898/2025.12.15.694023; this version posted December 16, 2025. The copyright holder for this preprint (which was not certified by peer review) is the author/funder, who has granted bioRxiv a license to display the preprint in perpetuity. It is made available under aCC-BY 4.0 International license.

Supplemental Figure 2:

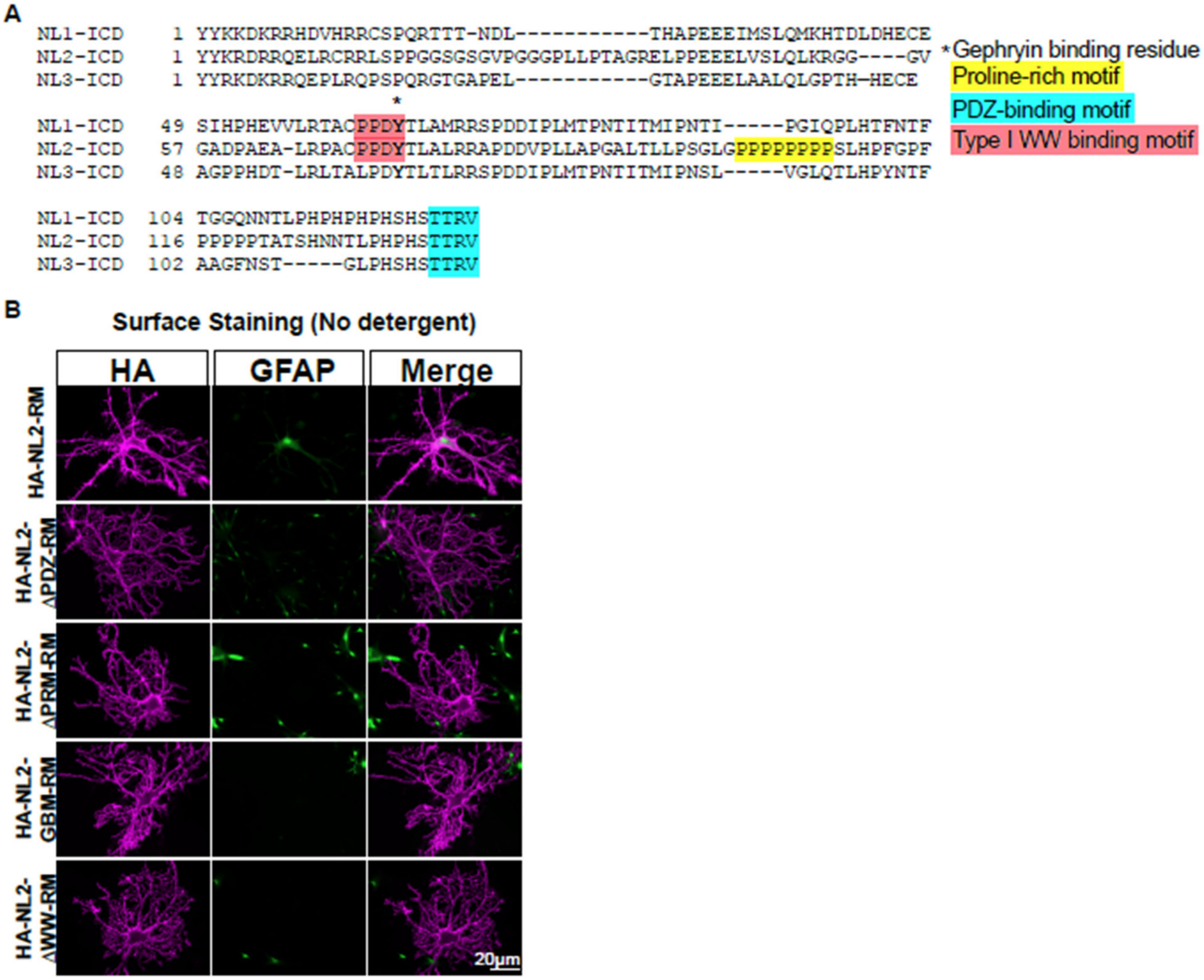

A) Mouse Neuroligin intracellular domain alignment, highlighting relevant protein sequence motifs.

B) Representative images of surface-stained astrocyte/neuron co-cultures. Cells were fixed with cold 4% PFA for 5 minutes, and triton was omitted from blocking steps to avoid intracellular staining. GFAP was used as a negative control. HA (magenta) signal shows membrane localization as expected. GFAP signal is largely absent in the absence of detergent.

bioRxiv preprint doi: https://doi.org/10.64898/2025.12.15.694023; this version posted December 16, 2025. The copyright holder for this preprint (which was not certified by peer review) is the author/funder, who has granted bioRxiv a license to display the preprint in perpetuity. It is made available under aCC-BY 4.0 International license.

1540 **Supplemental Figure 3:**

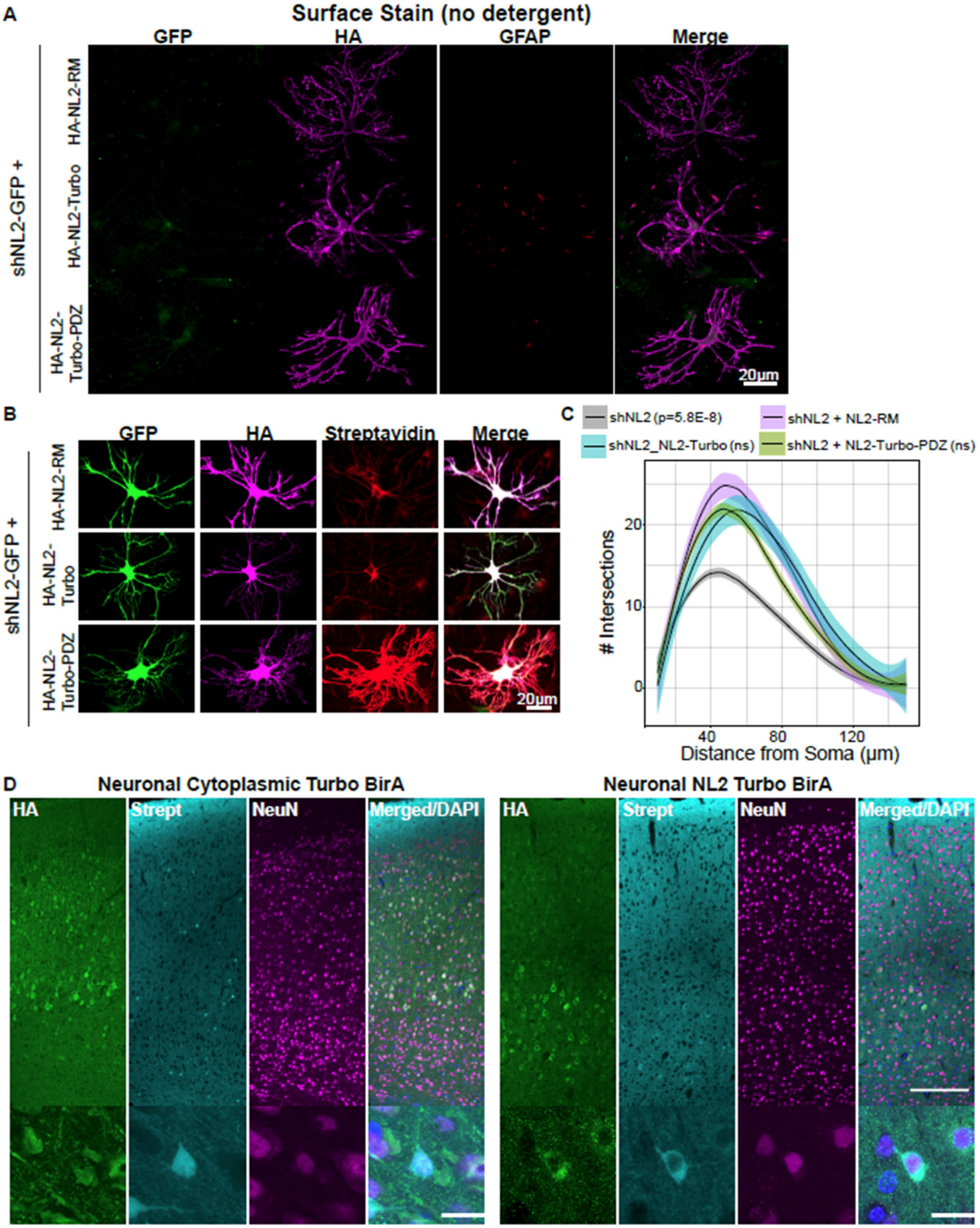

1541

bioRxiv preprint doi: https://doi.org/10.64898/2025.12.15.694023; this version posted December 16, 2025. The copyright holder for this preprint (which was not certified by peer review) is the author/funder, who has granted bioRxiv a license to display the preprint in perpetuity. It is made available under aCC-BY 4.0 International license.

A) Representative images of surface-stained astrocyte/neuron co-cultures. Cells were fixed with cold 4% PFA for 5 minutes, and triton was omitted from blocking steps to avoid intracellular staining. GFP and GFAP were used as negative controls. HA (magenta) signal shows membrane localization as expected.

B) Representative images demonstrating effective biotinylation (Streptavidin Alexa 594, red) in HA-NL2-Turbo-PDZ but not in HA-NL2 (negative control) or HA-NL2-Turbo. Experiments were performed at least twice with at least 20 cells per condition.

C) Sholl analysis quantification of images in B. Linear-mixed model reveals the main effect of condition, $F(1,3)=11.01$, $p=1.4E-6$. P-values represent Dunnett's post-hoc with respect to shNL2 + NL2-RM. NS = not significant in Dunnett's. N = 14-56 cells across 3 biological replicates (independent culture experiments).

D). Representative cortical images of Neuronal Turbo BirA injected animals. Stained with Streptavidin-594 (Strept), HA and NeuN to determine neuronal specificity. Scale bars: column image = 100μm; inset = 20μm.

bioRxiv preprint doi: https://doi.org/10.64898/2025.12.15.694023; this version posted December 16, 2025. The copyright holder for this preprint (which was not certified by peer review) is the author/funder, who has granted bioRxiv a license to display the preprint in perpetuity. It is made available under aCC-BY 4.0 International license.

Supplemental Figure 4:

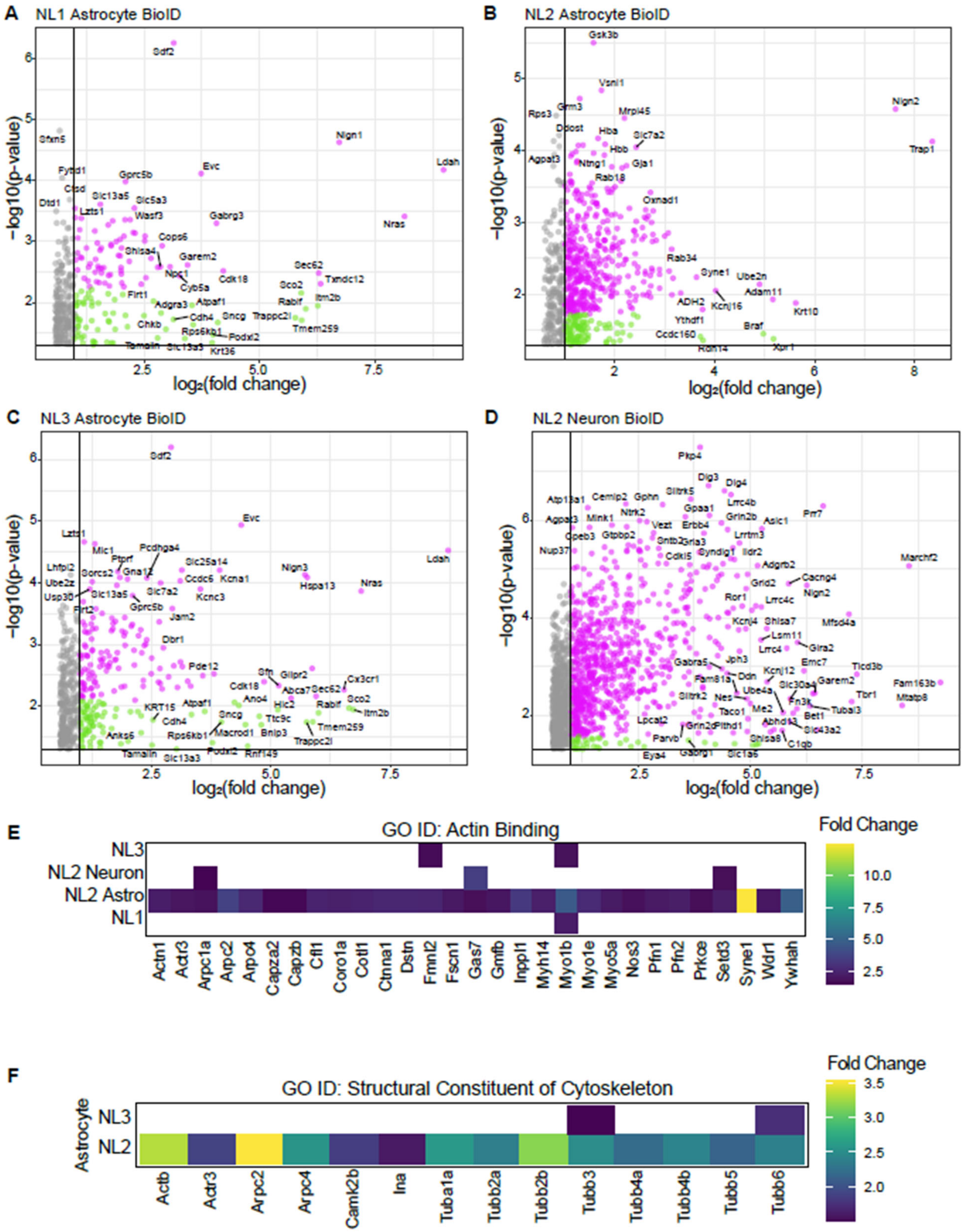

bioRxiv preprint doi: https://doi.org/10.64898/2025.12.15.694023; this version posted December 16, 2025. The copyright holder for this preprint (which was not certified by peer review) is the author/funder, who has granted bioRxiv a license to display the preprint in perpetuity. It is made available under aCC-BY 4.0 International license.

A-D) Volcano plots of all proteins identified in the iBioID experiments. Pink dots indicate proteins enriched over cytoplasmic control with a fold change > 1.5 and an FDR-corrected p-value < 0.05. Green dots indicate proteins enriched over cytoplasmic control with a fold change > 1.5 and a p-value < 0.05.

E-F) Expression of genes related to Actin Binding (E) or Structural Constituent of Cytoskeleton (F) from Gene Ontology analysis of iBioID shows that astrocytic NL2 significantly interacts with cytoskeletal genes, compared to astrocyte NL1/NL3 or neuronal NL2.

bioRxiv preprint doi: https://doi.org/10.64898/2025.12.15.694023; this version posted December 16, 2025. The copyright holder for this preprint (which was not certified by peer review) is the author/funder, who has granted bioRxiv a license to display the preprint in perpetuity. It is made available under aCC-BY 4.0 International license.

Supplemental Figure 5:

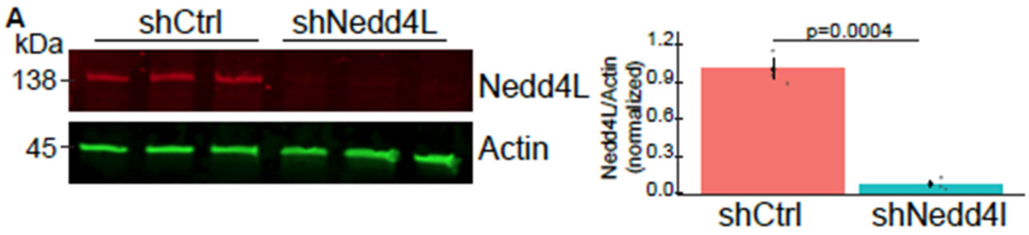

A) *left:* Western blots of astrocytes treated with lentivirus expressing shCtrl (scrambled shRNA) or shNedd4l. *Right:* Densitometry quantification [ImageJ]. The p value represents the results of the Student's t-test.

bioRxiv preprint doi: https://doi.org/10.64898/2025.12.15.694023; this version posted December 16, 2025. The copyright holder for this preprint (which was not certified by peer review) is the author/funder, who has granted bioRxiv a license to display the preprint in perpetuity. It is made available under aCC-BY 4.0 International license.

Supplemental Figure 6:

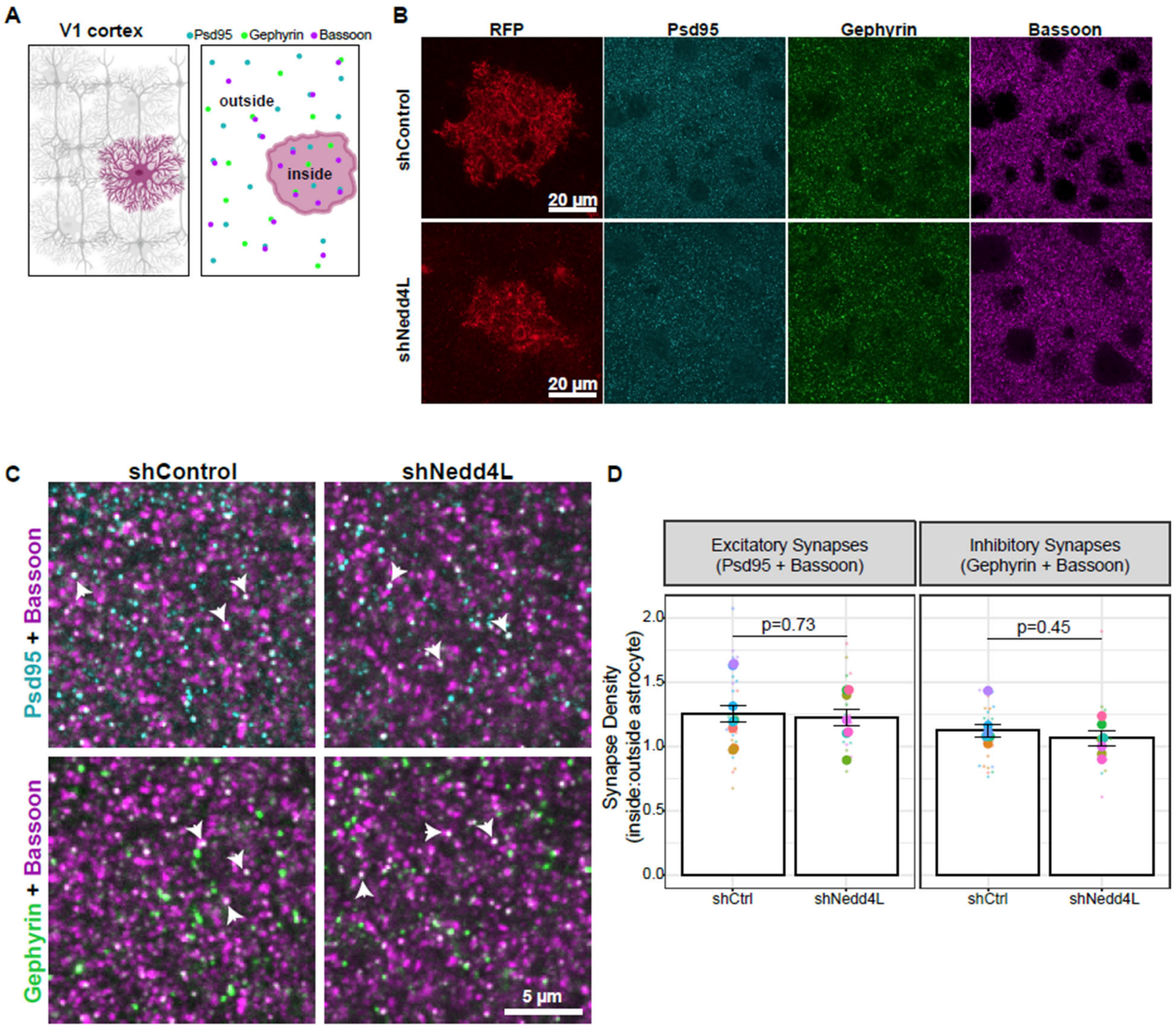

A) Cartoon of analysis. Sparsely labeled astrocytes (shControl mCherry CAAX; shNedd4l mCherry CAAX) were imaged in P21 mouse V1 cortex. Synapses (colocalized Psd95 + Bassoon [excitatory synapses] or Gephyrin + Bassoon [inhibitory synapses]) were quantified within the astrocyte territory (inside) and outside of the astrocyte territory (outside).

bioRxiv preprint doi: https://doi.org/10.64898/2025.12.15.694023; this version posted December 16, 2025. The copyright holder for this preprint (which was not certified by peer review) is the author/funder, who has granted bioRxiv a license to display the preprint in perpetuity. It is made available under aCC-BY 4.0 International license.

B) Representative images of mCherry-labeled astrocytes (RFP antibody), with Psd95 (cyan), Gephyrin (green), Bassoon (magenta) staining.

C) Magnified image showing colocalized synapse puncta for excitatory (Psd95 + Bassoon), and inhibitory (Gephyrin and Bassoon) synapses. Colocalized puncta are shown in white, with examples indicated with arrows.

D) Quantification of synapse density within labeled astrocyte to the outside unlabeled astrocyte territory. Large dots represent individual animals, with small dots representing each image from an animal. P-values indicate main effect of linear mixed effects ANOVA. Excitatory synapses: $F(1,13) = 0.12$, $p=0.73$. Inhibitory synapses: $F(1,13) = 0.61$, $p=0.45$. 7-8 animals were used per condition, with 4-8 images taken per animal.

bioRxiv preprint doi: https://doi.org/10.64898/2025.12.15.694023; this version posted December 16, 2025. The copyright holder for this preprint (which was not certified by peer review) is the author/funder, who has granted bioRxiv a license to display the preprint in perpetuity. It is made available under aCC-BY 4.0 International license.

Supplemental Figure 7:

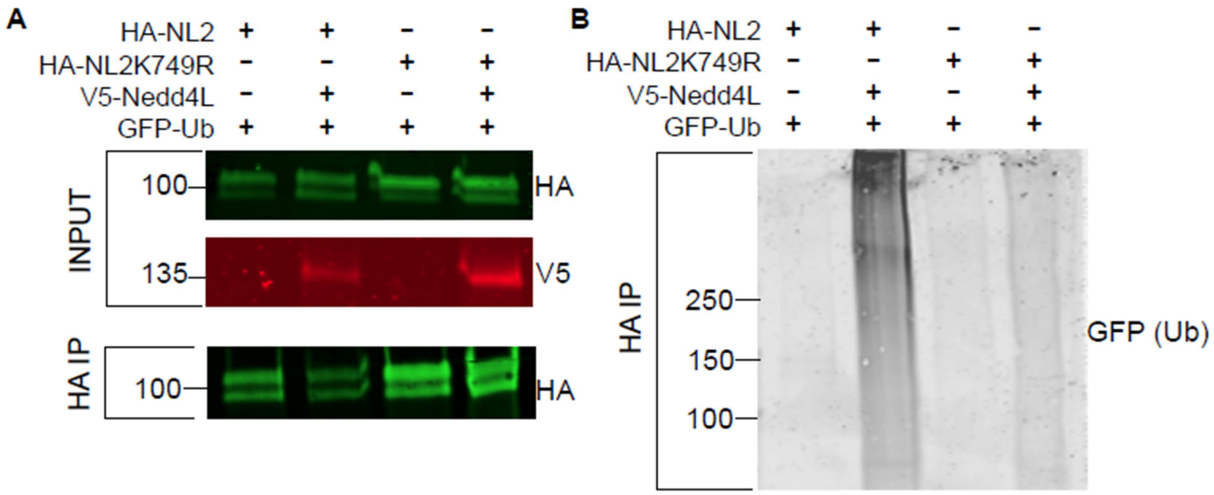

A, B) Western blot from HEK cell lysates and HA-NL2 IPs shows NL2 ubiquitination with Nedd4l overexpression, which is lost when NL2 is mutated at K749. GFP-tagged ubiquitin was overexpressed in these cells to visualize ubiquitinated NL2 in the immunoprecipitation.

bioRxiv preprint doi: https://doi.org/10.64898/2025.12.15.694023; this version posted December 16, 2025. The copyright holder for this preprint (which was not certified by peer review) is the author/funder, who has granted bioRxiv a license to display the preprint in perpetuity. It is made available under aCC-BY 4.0 International license.

Supplemental Dataset 1: In vivo BioID results. All proteins significantly enriched in Astrocytic NL1 (sheet 1), Astrocytic NL2 (sheet 2), Astrocytic NL3 (sheet 3), and Neuronal NL (sheet 4) iBioID compared to Astrocytic TurboID alone.

Supplemental Dataset 2: iBioID Gene Ontology output. All biological process gene ontology results. Column 1 indicates the iBioID list to which the categories correspond to.

Supplemental Table 1: Detailed statistical analyses for each figure panel.
